# Occurrence, Source Inference, and Risk Assessment of Per- and Polyfluoroalkyl Substances in Effluents, River Water and Groundwater from the Lijiang River Basin, a Typical Karst Region

**DOI:** 10.3390/toxics14070548

**Published:** 2026-06-24

**Authors:** Jiali Qian, Chengyou Ma, Qi Chen, Qiaoyan Wu, Litang Qin, Yanpeng Liang, Honghu Zeng

**Affiliations:** 1 Guangxi Key Laboratory of Environmental Pollution Control Theory and Technology, Guilin University of Technology, Guilin 541004, China; qjl2813@163.com (J.Q.); cq15805540733@163.com (Q.C.); wuqiaoyan1403@163.com (Q.W.); qinsar@glut.edu.cn (L.Q.); zenghonghu@glut.edu.cn (H.Z.); 2 College of Earth Sciences, Guilin University of Technology, Guilin 541004, China; machengyou2022@163.com; 3 University Engineering Research Center of Watershed Protection and Green Development, University of Technology, Guilin 541006, China; 4 Key Laboratory of Carbon Emission and Pollutant Collaborative Control, Education Department of Guangxi Zhuang Autonomous Region, Guilin University of Technology, Guilin 541006, China

**Keywords:** PFAS, karst hydrogeology, groundwater–surface-water interaction, positive matrix factorization, ecological and human health risk assessment

## Abstract

Research on the river-groundwater cross-contamination of per- and polyfluoroalkyl substances (PFAS) in karst regions is limited. We therefore investigated the PFAS occurrence, spatial distribution, sources and ecological risks in the Lijiang River basin, a typical karst area. PFAS concentrations were relatively low (0.08–74.0 ng/L, mean 4.13 ng/L). PFBA, PFHxA, PFNA and 6:2 FTS were widely detected. Short-chain PFAS concentrations (0.08–74.0, mean 4.75 ng/L) were higher than long-chain ones (0.02–3.31, mean 0.72 ng/L). Unusually, groundwater PFAS concentrations (0.08–74.0, mean 7.97 ng/L) exceeded those in rivers (0.08–11.7, mean 2.31 ng/L). Positive matrix factorization (PMF) combined with spatial distribution identified five main sources: sewage treatment plants (24.0%), gas station leaks/wastewater discharges (21.3%), untreated domestic sewage (18.1%), small-scale industrial wastewater (16.7%), and agricultural/aquaculture wastewater (20.2%). The ecological risk assessment showed that, except for PFUnDA posing a low risk to algae, the other PFASs presented no significant risk to algae, daphnia or fish. The human health risk assessment indicated minimal direct health risks. Our findings indicate that some PFASs in groundwater and river water may share common sources, highlighting the complex PFAS migration between rivers and groundwater in karst regions.

## 1. Introduction

Per- and polyfluoroalkyl substances (PFASs) exhibit environmental persistence and chemical stability due to their C-F bonds and microbial resistance, demonstrating multi-endpoint biological toxicity, including disrupted hepatic lipid metabolism and thyroid hormone imbalance [1,2,3,4,5]. The hydrophobic and oleophobic nature of PFASs enables their shorter-chain homologues to migrate extensively in aquatic environments [6]. With diverse contamination sources, PFASs are widely detected in rivers and groundwater globally, with concentrations ranging from ng/L to mg/L levels [7,8]. Generally, sediment-water partition coefficients (K_oc_) increase with PFAS chain length, favoring sediment retention over water distribution [9]. However, water quality parameters such as pH, electrical conductivity (EC), total dissolved solids (TDS), and salinity influence PFAS adsorption onto sediments [10]. Research indicates that tributary convergence and flow velocity (topography) may also influence the release of PFAS from sediments [5], thereby indirectly affecting PFAS concentrations in water. Furthermore, the impact of natural fluid dynamics, such as wind and waves, on contaminant concentrations has been extensively studied [11,12].

Karst regions typically feature well-developed pinnacle plains, karst depressions, and sinkholes on the surface [13]. Beneath the ground, complex and unique hydrogeological structures dominated by fissures, dissolution conduits, and caves form due to hydrochemical dissolution and physical erosion [13]. The Guilin Lijiang River basin in China exemplifies typical karst topography, featuring dense karst valleys. Its highly developed underground river system and pollution inputs driven by tourism make it a natural laboratory for studying karst environmental behavior under human intervention [14,15]. Consequently, conducting pollutant detection and analysis in karst water bodies is critically necessary. Recent frameworks in water ecology emphasize that systematic ecological assessments are fundamental to planetary sustainability, as they integrate water quality, ecosystem health, and anthropogenic stressors [16,17].

The inherent hydrogeological complexity of karst systems presents a significant challenge for traditional process-based or parameter-specific approaches to pollutant source apportionment. Such approaches would require an exhaustive and often impractical characterization of numerous hydrological and biogeochemical parameters (e.g., flow velocity, sorption kinetics, pH-dependent partitioning) across multiple scales to accurately predict PFAS fate and transport. The inherent uncertainty in parameterizing these processes in karst media can limit the robustness of source identification. Consequently, a holistic approach, such as receptor-based source apportionment, offers a pragmatic and effective alternative. Receptor models, including positive matrix factorization (PMF), Unmix models, and absolute principal component scores-multivariate linear regression (APCS-MLR), have frequently been employed in recent studies for source analysis [18]. Sheng et al. [18] employed the PMF model for the source apportionment of PFAS in rivers and groundwater, demonstrating good applicability in both scenarios. Li et al. [19] used PMF to trace PFASs in rivers, identifying agricultural chemicals, pharmaceuticals, and the textile industry as primary sources accounting for 38.1% of total concentrations; contributions from household pollution, tanning, and coating materials had increased annually, while proportions from electrochemical fluorination and chemical recycling have steadily decreased. Currently, PMF is widely applied for PFAS source apportionment in aquatic environments, but its use in karst regions remains limited.

Given this, this study (i) employs ultra-performance liquid chromatography–mass spectrometry/mass spectrometry (UPLC-MS/MS) (ACQUITY UPLC I-Class/Xevo TQ-S micro, Waters Corporation, Milford, MA, USA) to analyze the contamination levels of 24 target PFAS and their spatial distribution patterns in river water and groundwater from typical karst regions; (ii) investigates factors potentially influencing PFAS concentration distribution patterns in karst river water and groundwater; (iii) infers PFAS sources using the PMF model combined with actual site information; and (iv) assesses the ecological risks and potential health risks of the major detected PFAS. This study contributes to a better understanding of the current water quality status and PFAS pollution in rivers and groundwater within karst regions. It identifies the specific patterns of PFAS contamination in water bodies within these karst areas, providing crucial data support for water environment management.

## 2. Materials and Methods

### 2.1. Study Area and Sampling

**Figure 1:**
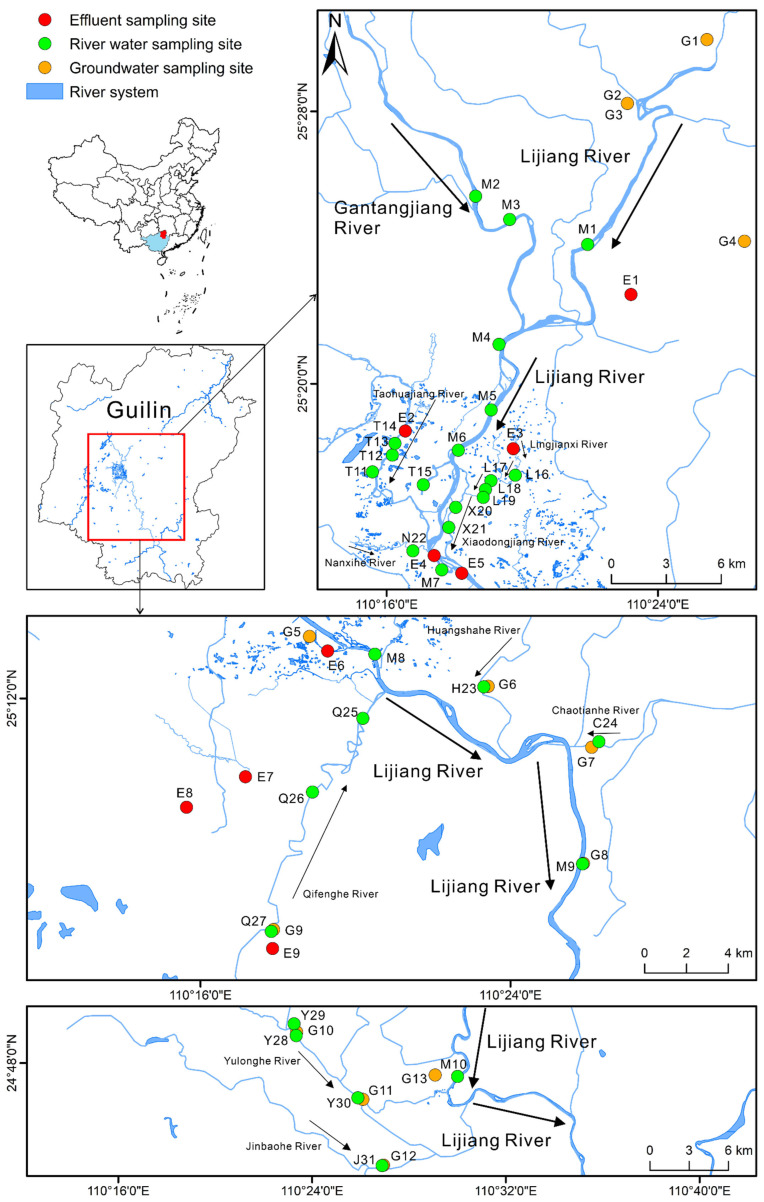
Sampling point distribution and PFAS spatial distribution map; arrows denote river flow directions.

Sampling was conducted in July 2023. At each sampling point, three field replicate samples were collected. For all subsequent analyses, the measured PFAS concentrations from the three field replicates were averaged to represent a single value for that sampling site, and all results are therefore reported per sampling site (*n* = 53). The distribution map of sampling points is shown in Figure 1. Around the Lijiang River basin, nine points (E1–E9) were established as effluent sampling points: downstream of WWTPs, near hospitals and industrial parks, and along rivers receiving livestock wastewater discharges. River water sampling points covered the main and tributary streams of the Lijiang River basin, totaling 31 sampling points. Thirteen groundwater sampling points (G1–G13) were established to assess the pollution impacts from agricultural activities and aquaculture on groundwater. Detailed information on the sampling points is provided in Table S1. Samples were collected and preserved according to routine water quality indicators and PFAS testing requirements during sampling. The specific sampling procedures are shown in Text S1.

### 2.2. Sample Pretreatment and Analysis

The collected water samples underwent pretreatment, as detailed in Text S2. The method’s feasibility and matrix effects were evaluated through spiked blank recovery tests, yielding average recovery rates ranging from 76.7% to 115% for 24 PFAS analytes across three parallel samples. To minimize potential PFAS contamination, samples were collected and stored following strict protocols (Text S1), and all materials in contact with the samples were pre-screened to avoid fluoropolymer-containing items. This study employed ultra-performance liquid chromatography–mass spectrometry/mass spectrometry (UPLC-MS/MS) (ACQUITY UPLC I-Class/Xevo TQ-S micro, Waters Corporation, Milford, MA, USA) to analyze the samples using the external standard method. Detailed instrument parameters are shown in Text S3 and the details of the 24 target PFAS standards, chemical reagents, and experimental instruments are provided in Tables S2–S5. The elution programs and mass spectrometry conditions are detailed in Tables S6 and S7.

### 2.3. Quality Assurance/Quality Control

The limit of detection (*LOD*) for the target analytes is defined as three times the signal-to-noise ratio (S/N), and the limit of quantification (LOQ) is defined as ten times the S/N. For every ten samples, one duplicate, one blank, and one spiked blank sample are analyzed. A mixed standard solution of PFASs was prepared with concentration gradients of 0.1, 0.5, 1, 5, 10, 20, 100, and 200 μg/L to establish the calibration curve. The PFASs demonstrate strong linearity within the 0.1–200 μg/L concentration range, with a linear correlation coefficient (*R*^2^) greater than 0.99. The detection limits (DL) and quantification limits (QL) for the PFASs range from 0.01 to 0.95 ng/L and 0.03 to 3.17 ng/L, respectively. These values are substantially lower than the highest concentration in the standard solution, reflecting the high sensitivity of the analytical method. Twenty-four PFASs frequently detected in the environment were selected as target PFASs. Of these, 12 were detected: PFBA, perfluoropentanoic acid (PFPeA), PFHxA, perfluoroheptanoic acid (PFHpA), PFOA, PFNA, PFDA, PFUnDA, perfluorobutanesulfonate (PFBS), perfluoropentanesulfonate (PFPeS), perfluorohexanesulfonate (PFHxS), and 6:2 FTS, each with recovery rates exceeding 60%. Detailed information on the correlation coefficients, detection limits, and quantification limits for the 24 PFAS compounds is provided in Table S8. The Organization for Economic Cooperation and Development (OECD) regulations classify PFAS with six or more fully fluorinated carbon atoms as long-chain PFAS. Despite containing six fully fluorinated carbon atoms, 6:2 FTS is frequently considered a novel short-chain alternative.

### 2.4. PMF Model

PMF excels at analyzing mixed data from various sources and accurately quantifies the contribution of each component [20]. The fundamental principle of the model assumes that an environmental sample *X* is an *n* × *m* matrix, where *n* represents the number of samples and *m* represents the number of chemical components. *X* can then be decomposed into a source contribution matrix *G* (*n* × *p*, where *p* is the number of sources), a source component matrix *F*(*p* × *m*), and a residual matrix *E*(*n* × *m*). The measured sample concentration can be expressed as [21]:

(1) $$X_{\left(n\times m\right)}=G_{\left(n\times p\right)}F_{\left(p\times m\right)}+E_{\left(n\times m\right)}$$

First, values below the limit of detection (*LOD*) should be replaced with 1/2 *LOD*. Then, for values below the *LOD*, the uncertainty (*U_nc_*) is calculated using the formula:

(2) $$U_{\mathrm{nc}}=5/6\mathrm{LOD}$$

For data exceeding the *LOD*, *U_nc_* uses the formula:

(3) $$U_{\mathrm{nc}}=\sqrt{{\left(\mathrm{Error}\;\mathrm{Fraction}\times \mathrm{concentration}\right)}^{2}+{\left(0.5\times \mathrm{LOD}\right)}^{2}}$$

Among these, the *Error Fraction* represents the experimental error value, typically ranging between 0.05 and 0.2. Finally, the PMF model is run with the data and uncertainty, debugging via the S/N ratio and *R*^2^ (requiring an *R*^2^ value greater than 0.6) to ensure that Q (True)/Q (robust) remains below 1.5, thereby determining the appropriate number of factors.

### 2.5. Risk Assessment

#### 2.5.1. Ecological Risk Evaluation

The primary objective of ecological risk assessment is to predict the likelihood of various environmental pollutants causing harm to ecosystems or one or more key components within them. Target PFAS compounds were screened and organized based on their toxicological data across three trophic levels: algae, daphnia, and fish. Specific parameters are detailed in Table S9 [22]. To understand the ecological risks of PFASs in the study area’s aquatic environment, the risk quotient (*RQ*) was employed to assess the ecological toxicity risk of PFASs in river water within the Lijiang River. The *RQ* is defined as the ratio of the measured environmental concentration (*MEC*) and the no-observed-effect concentration (*NOEC*) or predicted no-effect concentration (*PNEC*) [23].

(4) $$\mathrm{RQ}=\frac{\mathrm{MEC}}{\mathrm{PNEC}}$$

where *RQ* denotes the risk quotient, dimensionless; *MEC* denotes the measured environmental concentration, ng/L; and *PNEC* denotes the predicted no-effect concentration, ng/L.

(5) $$\mathrm{PNEC}=\frac{{\mathrm{LC}}_{50}}{1000}or\frac{{\mathrm{EC}}_{50}}{1000}$$

where *LC*_50_ denotes the short-term median lethal concentration, ng/L; *EC*_50_ denotes the median effective concentration, ng/L; *PNEC* denotes the predicted no-effect concentration for aquatic organisms for each compound, ng/L; 1000 represents the value of the assessment factor for acute toxicity data.

Generally, ecological toxicity risk assessments are categorized into four levels based on the *RQ* value: high risk (*RQ* > 0.50), medium risk (0.10 < *RQ* < 0.50), low risk (0.01 < *RQ* < 0.10), and no risk (*RQ* < 0.01) [24].

#### 2.5.2. Health Risk Evaluation

The hazard quotient (*HQ*) model is commonly used to assess the health risks of PFAS in various environmental media [25]. Health risks were estimated for individuals of different ages and genders, categorized into seven groups: 3–6 years, 7–11 years, 12–16 years, 17–19 years, 20–24 years, 25–59 years, and over 60 years. The level of human health risk was calculated using the following formula for the *HQ* value.

(6) $$\mathrm{HQ}=\frac{\mathrm{MEC}}{\mathrm{DWEL}}$$

where *HQ* denotes the PFAS hazard quotient; *MEC* represents the measured environmental concentration of PFASs in river water samples (ng/L); and *DWEL* indicates the drinking water equivalent level of PFASs (ng/L).

(7) $$\mathrm{DWEL}=\frac{P\times \mathrm{ADI}\times \mathrm{BW}}{\mathrm{DWI}\times \mathrm{AB}\times \mathrm{FOE}}$$

where *DWEL* denotes the equivalent value of contaminants per liter of drinking water (μg/L); *P* represents the percentage of PFAS intake via drinking water, set at 0.2; *ADI* indicates the acceptable daily intake of PFASs for the seven age groups [μg/(kg·d)] (Table S10); *BW* denotes the average body weight (kg); *DWI* represents the drinking water intake (L/d) for the seven age groups (see Table S11); *AB* is the gastrointestinal absorption rate, assumed as 1; *FOE* is the exposure frequency (350 days/year), calculated as 0.96. Detailed *DWEL* calculation results are presented in Table S12.

PFAS typically exist in aquatic environments as mixtures, making it necessary to consider their combined toxicity when assessing risks to human health. *HQ_mix_* serves as the cumulative hazard quotient measure for PFAS, calculated using the following formula:

(8) $${\mathrm{HQ}}_{\mathrm{mix}}=\sum {\mathrm{HQ}}_{i}$$

The *HQ* value calculation results are presented in Table S13. Generally, the level of health risk assessment can be categorized into three tiers based on the *HQ* value: PFAS pose no risk or negligible health risks to humans (*HQ* < 0.2); PFASs have uncertain effects on human health, requiring further research (0.2 ≤ *HQ* < 1.0); and PFASs pose significant adverse effects on human health (*HQ* ≥ 1.0) [26,27].

### 2.6. Statistical Analysis

This study drew PFAS concentration level maps using Origin 2021 (Origin Lab, Northampton, MA, USA), generated the maps of sampling point distribution and PFAS spatial distribution using ArcGIS 10.2 software (ESRI, Redlands, CA, USA), conducted redundancy analysis using R Studio 4.5 (RStudio, Inc., Boston, MA, USA), and conducted a source tracing analysis of PFASs in river water and groundwater using PMF 5.0 software (U.S. EPA, Washington, DC, USA). Correlation analysis was performed using Pearson correlation analysis with a significance level of *p* < 0.05 (two-tailed), and other data analyses were conducted using statistical software SPSS version 25 (IBM, Armonk, NY, USA) and Microsoft Excel 2021 (Microsoft Corporation, Redmond, WA, USA).

## 3. Results and Discussion

### 3.1. PFAS Concentration LEVELS

Water samples were collected from nine effluents, 31 river sites, and 13 groundwater sites. Of the 24 target PFAS compounds, 12 were detected in at least one sample (Table S14), including PFBA, PFPeA, PFHxA, PFHpA, PFOA, PFNA, PFDA, PFUnDA, PFBS, PFPeS, PFHxS, and 6:2 FTS. Overall, compared to the PFAS detection levels reported domestically and internationally (Table 1), the total PFAS concentrations (∑PFAS) in the 53 water samples across different categories were relatively low (0.08–74.0, avg. 4.13 ng/L). Groundwater ∑PFAS concentrations (0.08–74.0, 7.97 ng/L) were higher than those in effluents (0.78–21.9, 4.83 ng/L) and river water (0.08–11.7, 2.31 ng/L). Short-chain ∑PFAS concentrations (0.08–74.0, 4.75 ng/L) were higher than long-chain ∑PFAS concentrations (0.02–3.31, 0.72 ng/L) across all three water bodies, with short-chain PFASs accounting for as much as 86.9% of the total. In contrast to traditional pollution dominated by long-chain PFOA and PFOS, PFAS contamination in the Lijiang River basin is primarily associated with short-chain PFASs, which aligns with the international trend of short-chain PFASs gradually replacing long-chain variants. Among the short-chain PFASs, PFBA, PFHxA, and 6:2 FTS were the primary detected species, each with detection frequencies exceeding 60% (Tables S15–S17). For long-chain PFASs, PFNA contributed the highest concentrations across all three water bodies. This may result from the increasing hydrophobicity of PFASs with chain length, which enhances lipophilicity and promotes sediment partitioning. Consequently, PFNA (C = 9) is more readily detected in water bodies than other long-chain PFAS (C > 9) [28,29].

#### 3.1.1. Effluent

**Figure 2:**
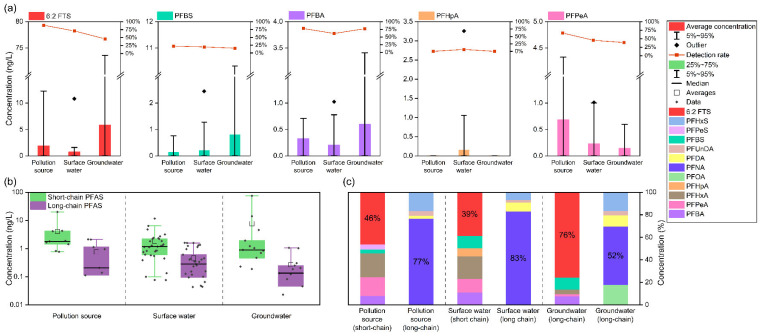
(**a**) PFAS concentration levels and detection rates in effluent, river water and groundwater (PFASs with maximum detected concentration ≥ 3 ng/L); (**b**) quartile plots of long-chain PFASs and short-chain PFAS concentrations in effluent, river water and groundwater; and (**c**) percentage distribution plots.

Ten PFASs were detected in the four effluents, including PFBA, PFPeA, PFHxA, PFNA, PFDA, PFUnDA, PFBS, PFPeS, PFHxS, and 6:2 FTS, with the concentrations of ∑PFAS ranging from 0.78 to 21.9 ng/L, 4.83 ng/L (see Figure 2a and Table S15). Short-chain PFAS concentrations remained predominant (84.9%), with concentrations (0.78–19.8, 4.10 ng/L) higher than those of long-chain PFASs (0–2.13, 0.73 ng/L) (see Figure 2b). Among the short-chain PFASs, the primary PFASs were 6:2 FTS (0.13–12.3, 1.89 ng/L), PFHxA (0.09–2.81, 0.87 ng/L), PFPeA (0.66–4.33, 0.68 ng/L), and PFBA (0.25–0.71, 0.33 ng/L), with detection rates of 88.9%, 100%, 66.7%, and 77.8%, respectively. Among the long-chain PFASs, the primary PFASs were PFNA (0.10–1.60, 0.58 ng/L), contributing 77.0%. Among the four effluent types, the ΣPFAS concentration in wastewater from WWTPs (E2, E5, E6, and E9; 1.96–21.9, avg. 6.89 ng/L) was similar to that in hospital wastewater (E3 and E4; 4.50–6.31, 5.41 ng/L), with the ΣPFAS concentrations of the other effluents all being below 3 ng/L (see Table S14).

**Figure 3:**
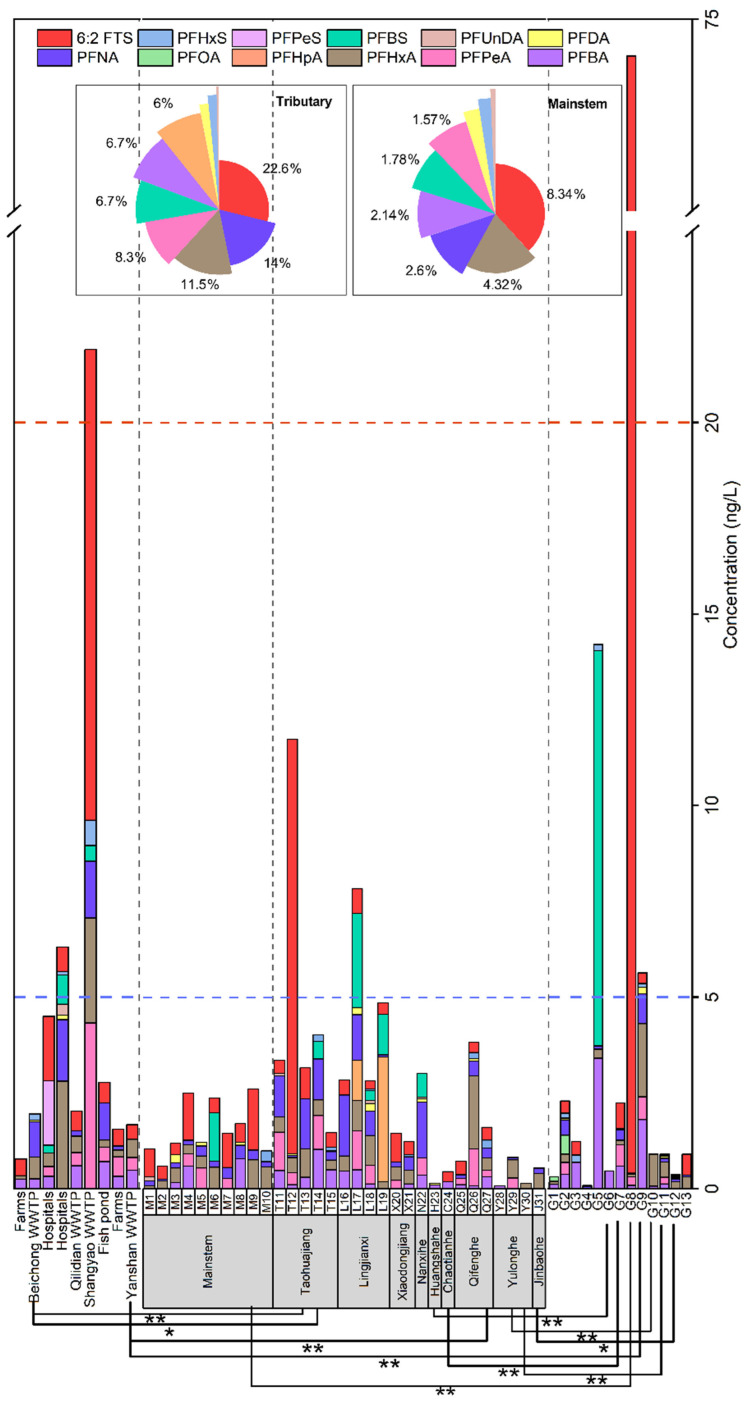
Stacked column chart of PFAS concentrations in the 53 sampling sites, the proportion of different PFASs in the main and tributary streams of the Lijiang River, and the correlation of PFASs in the sampling sites close to each other (“**” indicates that the correlation is significant at the 0.01 level; “*” indicates that the correlation is significant at the 0.05 level).

Figure 3 shows that only the ΣPFAS concentration downstream of the WWTP (E6; 2.19 ng/L) was higher than those at the upstream sampling point (E5; 0.20 ng/L) and the river water sampling point (M8; 0.17 ng/L). This finding demonstrates that the WWTP substantially influences PFAS concentrations in surrounding rivers. WWTPs are a primary source of PFASs in aquatic environments. Conventional wastewater treatment processes are generally ineffective at removing PFAS, often resulting in elevated concentrations at plant effluent points [51,52,53]. Additionally, precursor transformations and PFAS solid–liquid partitioning characteristics contribute to elevated short-chain PFAS concentrations in WWTP (E6, 1.98 ng/L; E5, 0.19 ng/L; M8, 0.13 ng/L) [54,55,56,57]. Other WWTPs exhibited ΣPFAS concentrations (0.04–4.33, 0.19 ng/L) similar to those in surrounding rivers (M7 and Q27, 0.18–0.90, 0.15 ng/L), suggesting that PFASs in these effluents primarily originate from domestic sewage. The average daily wastewater treatment capacity of the plants in the study area is approximately 68,500 tons, resulting in a potential daily PFAS mass flow of 472 mg, which imposes a measurable burden on the river.

As shown in Table S14, the ∑PFAS concentration in wastewater near the hospital (4.50–6.31, 5.41 ng/L) was slightly higher than that in river water (0.08–11.7, 2.31 ng/L). Hospitals are in areas with high levels of human activity, discharging large volumes of domestic wastewater daily. Studies indicate that rivers flowing through densely populated urban areas are impacted by human production activities, leading to increased pollutant loads in rivers [58]. For example, sampling sites in the densely populated St. Lawrence Valley in Quebec, Canada, exhibited higher PFAS concentrations, whereas sites in the less developed eastern region showed significantly lower levels [59]. Thus, PFASs in medical wastewater still contribute to domestic sewage loads.

Since the effluent in the study area primarily consists of domestic sewage, the overall PFAS concentration levels at the sampling sites were low (0.78–21.9, 4.83 ng/L), lower than levels typically reported in contaminated water bodies or polluted source discharges [33,34,36]. Short-chain PFASs accounted for a greater proportion than long-chain PFASs in these effluents. Both short-chain and long-chain PFASs were detected in effluent from WWTPs in Tianjin and Guangzhou of China and in USA [30,31,35,36].

#### 3.1.2. River Water

Ten PFASs were detected in 31 river water samples, including PFBA, PFPeA, PFHxA, PFHpA, PFNA, PFDA, PFUnDA, PFBS, PFHxS, and 6:2 FTS, with the concentrations of ∑PFAS ranging from 0.08 to 11.7, 2.31 ng/L (see Figure 2a and Table S16). Short-chain PFAS concentrations remained predominant (74.0%), with concentrations (0.08–11.6, 1.71 ng/L) higher than those of long-chain PFASs (0–3.31, 0.60 ng/L) (see Figure 2b). The main substances detected were 6:2 FTS (0.13–10.8, 0.72 ng/L), PFNA (0.04–1.59, 0.38 ng/L), and PFHxA (0.06–1.90, 0.37 ng/L), with detection frequencies of 74.2%, 90.3%, and 74.2%, respectively. Similarly, in Biscayne Bay, the detection rate of the short-chain PFAS substitute 6:2 FTS was 53% [45]; 161 U.S. streams are also experiencing the coexistence of short-chain and long-chain PFASs [39]. In Shanghai, China, PFAS concentrations of C4–C7 were higher than those of C9–C14 in water bodies near fluorine-related industrial zones and forest parks. Short-chain PFAS substitution was also prevalent in these river waters [41].

PFAS concentrations in the river water of the Lijiang River basin are relatively low (0.08–11.7, 2.31 ng/L), lower than those in eastern Mediterranean river water (0–13,400, 4030 ng/L) [42], Biscayne Bay river water (0.17–51.0, 357 ng/L) [45], and the Beiluo and Qingjian Rivers in the Yellow River basin (4.28–372, 50.1 ng/L) [60]. The main source of water pollution is domestic sewage inflow.

#### 3.1.3. Groundwater

Ten PFASs were detected in 13 groundwater samples, including PFBA, PFPeA, PFHxA, PFOA, PFNA, PFDA, PFUnDA, PFBS, PFHxS, and 6:2 FTS, with the concentrations of ∑PFAS ranging from 0.08 to 74.0, 7.97 ng/L (see Figure 2a and Table S17). Short-chain PFAS concentrations remained predominant (96.7%), with concentrations (0–74.0, 7.70 ng/L) higher than those of long-chain PFASs (0–1.07, 0.27 ng/L). As long-chain PFASs are phased out from industrial products, short-chain PFASs are increasingly used as substitutes for PFOA [61]. For instance, the detection rates of short-chain PFASs exceeded those of long-chain PFASs in a Korean river and groundwater in Fuxin City, Liaoning Province [62,63]. The primary detected substances in groundwater were 6:2 FTS (0.13–73.6, 5.83 ng/L), PFBS (0.02–10.3, 0.80 ng/L), and PFBA (0.06–3.41, 0.60 ng/L), with detection frequencies of 46.2%, 15.4%, and 76.9%, respectively. Within the karst terrain of the Lijiang River basin, PFAS concentrations in groundwater exceed those in the river water bodies. This concentration inversion is largely governed by the distinctive hydrogeological properties of the karst region.

The PFAS concentration levels in groundwater within the Lijiang River basin are relatively low (0.08–74.0, 7.97 ng/L), lower than those found in groundwater surrounding Canadian airports (0–10,800, 447 ng/L) [64], groundwater in California (0–5,180,000, 2440 ng/L) [46], the Maozhou River basin (9.90–592, 170 ng/L) [65], and Jiangsu Province (2.69–556, 43.1 ng/L) [66]; but higher than those in groundwater in the Jinjiang River basin (0.26–23.1, 6.25 ng/L) [67] and groundwater in the Beiluo River and Qingjian River basins of the Yellow River basin (0–14.7, 2.56 ng/L) [60].

### 3.2. Spatial Distributions of PFAS

#### 3.2.1. River Water

Among the river water sampling points, T12 exhibited the highest ∑PFAS (11.7 ng/L) due to its proximity to numerous residential areas and the impact of domestic sewage and industrial effluent on the Taohuajiang River, with the novel PFAS substitute 6:2 FTS accounting for a significant proportion (10.8 ng/L). Concentrations at sampling point L17 were the second highest (7.83 ng/L) due to domestic sewage and nearby industrial impacts. Tributaries like the Jinbao River and Yulong River flow through sparsely populated areas, resulting in low PFAS concentrations (<1 ng/L). ∑PFAS at all other river water sampling points remained below 5 ng/L.

PFAS levels in the mainstem of the Lijiang River (0.59–2.60, 0.56 ng/L) were lower than those in its tributaries (0.08–11.7, 2.67 ng/L). The percentage distribution of PFAS concentrations differed slightly between the main and tributary streams, with 6:2 FTS accounting for the largest proportion in both, followed by PFHxA (see in Figure 3). Tributaries cover a broader area, with most flowing through densely populated regions characterized by high human activity intensity, such as villages, sewage treatment plants, clinics, and farmlands. Due to the discharge of domestic sewage, medical wastewater, agricultural runoff, and industrial effluents, PFAS concentrations in tributaries were markedly elevated compared to those in the mainstem [51,52,53,54,68].

Near sampling points such as M1 and T12, there are agricultural fields and orchards. PFAS polymers are widely used in pesticide synthesis, and orchards and vegetable plots may employ pesticides, fertilizers, or soil conditioners containing PFAS. These substances can enter nearby water bodies via surface runoff, leading to elevated PFAS concentrations [68,69]. For instance, a study analyzing 80 PFAS compounds in sludge, compost, and chemical fertilizers from Quebec, Canada, detected PFASs in all samples [70].

#### 3.2.2. Groundwater

The PFAS concentrations detected at sampling points G5, G8, and G9 were higher than those at the other points, with ∑PFAS of 74.0 ng/L, 14.3 ng/L, and 6.30 ng/L, respectively (see Figure 3). PFBS was detected only at sampling points G5 and G12, with the highest concentration observed at G5 (10.3 ng/L). Sampling point G5 is located near fishponds and farmland. As relatively enclosed water bodies, fishponds are more susceptible to accumulating PFAS pollutants if contaminated sources enter them. For instance, a study on fishponds in Guangdong Province, China, showed significantly elevated PFAS concentrations in ponds near industrial zones, attributed to electroplating plant wastewater leakage and feed additive accumulation [71].

The highest concentration of 6:2 FTS was observed at the G8 sampling point, measuring 73.6 ng/L. The presence of karst topography, characterized by caves and fissures, has facilitated accelerated leakage from underground fuel storage tanks or pipelines at upstream gas stations. This process has resulted in persistent groundwater contamination by petroleum hydrocarbons and additives, including PFAS residues from firefighting foams [72]. As a short-chain PFOS substitute, 6:2 FTS serves as a primary component of aqueous film-forming foam (AFFF). Its high mobility contributes to the continued degradation of groundwater environments. Previous studies have identified AFFF residues in water bodies and fish populations up to 8 km from pollution sources [73]. Furthermore, certain PFASs may undergo photolysis in river water. For instance, 6:2 FTS can be converted into shorter-chain PFCAs, such as PFPeA and PFHxA, in microbiologically active soils or water bodies [61,74,75]. PFAS concentrations at sampling point G9 showed significant correlation with those at the nearby E9 sampling point (see Figure 3), suggesting that PFAS at G9 may be influenced by effluent from the WWTP. Karst landscapes are characterized by unique hydrological features. Their highly permeable structure enables pollutants to enter groundwater directly, creating a specific contamination pathway: point source to fissure to groundwater.

### 3.3. Correlation Relationship Analysis

**Figure 4:**
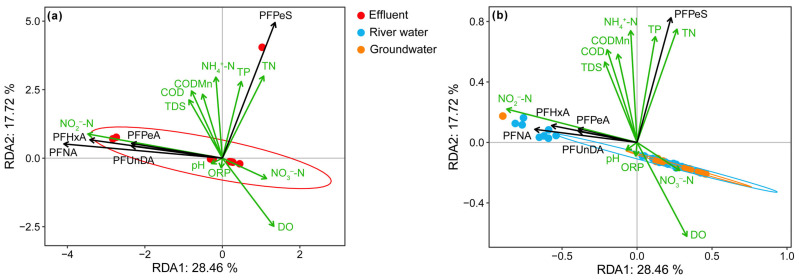
RDA of PFAS and environmental water quality parameters was conducted based on PFAS contamination levels in different water bodies: (**a**) effluents, (**b**) river water and groundwater. Arrow length indicates relationship strength, while arrow angles reveal correlation types between variables. The ellipses in the figure represent the 95% confidence ellipses for the samples.

Pearson correlation (see Figures S1–S3) and redundancy analysis (RDA) (see Figure 4) were performed to evaluate associations between environmental parameters (see Tables S18–S20) and PFASs across 9 effluents, 31 river water samples, and 13 groundwater samples. The results showed that environmental parameters accounted for 52.3% of the observed variance, and DO, TDS, NO_2_^–^-N, NH_4_^+^-N, and CODMn demonstrated strong correlations with PFAS concentrations (*p* < 0.01) (see Table S21).

DO showed significant negative correlations with most PFASs (PFPeA, PFHxA, PFBS, PFPeS, PFNA, and PFDA) (*R*^2^ = 0.29–0.71, *p* < 0.01). Tang et al. [63] examined the relationship between PFAS and DO in groundwater at a Chinese industrial park. Their study identified a significant negative correlation between PFASs and DO. Furthermore, the negative correlation observed in karst groundwater suggests that low-oxygen conditions may be associated with higher PFAS levels. When surface water reaches the subsurface through sinkholes, it is highly likely to become trapped within fractures or cavities. The DO levels in these stagnant water layers are extremely low, making PFASs susceptible to adsorption and accumulation by sediments. The secondary release of adsorbed PFASs can then cause a sharp increase in PFAS concentrations within the water. The pH exhibited a significant positive correlation with long-chain PFASs such as PFNA and PFDA (*R*^2^ = 0.28–0.35, *p* < 0.01). Campos et al. [76] reported that higher pH values enhance negative surface charges, increasing electrostatic repulsion and reducing PFAS adsorption, which promotes migration from solid to liquid phases. In contrast, short-chain PFASs are less sensitive to pH variations [77]. TOC was significantly positively correlated with several PFASs (PFBA, PFPeA, PFNA, PFBS, and PFPeS) (*R*^2^ = 0.25–0.44, *p* < 0.01). Additional studies have found strong associations between PFAS and organic matter indicators in soil and water. The adsorption of short-chain PFAS is primarily influenced by low-molecular-weight fulvic acids, potentially through co-metabolic processes, while organic matter also modulates PFAS adsorption and migration [78]. Gallen et al. [79] observed weak to moderate correlations between PFAS concentrations and both pH and TOC in Australian landfill leachate.

NO_2_^−^-N showed significant positive correlations with most PFASs (PFPeA, PFHxA, PFNA, PFDA, PFUnDA, PFBS, PFHxS, and 6:2 FTS) (*R*^2^ = 0.25–0.90, *p* < 0.01). NH_4_^+^-N, TN, and TP also exhibited strong correlations with specific PFASs (PFHpA, PFNA, PFBS, and PFPeS) (*R*^2^ = 0.25–0.98, *p* < 0.01). Similar moderate to strong correlations between PFAS concentrations and TN or NH_4_^+^-N have been reported in Chinese landfill leachate [80]. PFASs disrupt denitrifying enzyme activity. This inhibition suppresses gene expression in denitrifying bacteria. As a result, the conversion of NO_2_^−^-N/NO_3_^−^-N is blocked, causing an increase in TN [81]. Given the complexity of PFAS exposure in aquatic environments, further research is warranted to elucidate the interactions between environmental parameters and PFASs.

### 3.4. Source Analysis

**Figure 5:**
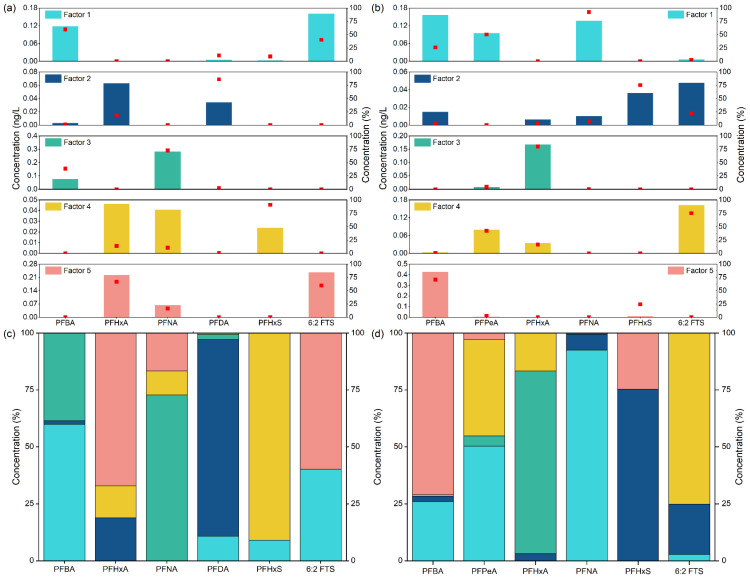
Basic factor profiles for different sources of PFAS in (**a**) river water and (**b**) groundwater as shown by the PMF model; percentage contribution of PMF factors to PFAS in (**c**) river water and (**d**) groundwater. The small red squares represent the percentage contribution values corresponding to the right vertical axis (%).

To identify major PFAS sources in the study area, we utilized the PMF model for concentration analysis. The model exhibited strong correlations between observed and predicted concentrations (*R*^2^ > 0.99 for river water and *R*^2^ > 0.85 for groundwater), indicating a good overall fit. A five-factor solution was selected after 20 model runs, producing a calibrated Q-value of 246.8 for river water (approximating the theoretical Q-value of 231.4) and a calibrated Q-value of 7.10 for groundwater, which was consistent with its theoretical Q-value. The results are presented in Figure 5 and Tables S22–S25. The DISP error estimation returned a first-order value of zero, confirming the validity of the PMF operation. To further evaluate the stability and independence of the resolved factors, a bootstrap analysis was performed. The results showed that the correlation coefficients between each base factor and its corresponding bootstrap factor were all greater than 0.80, while correlations between a given base factor and other bootstrap factors were negligibly low. These findings indicate that the five factors are stable, independent sources, and that the model results are reliable.

River water factor 1 accounts for 20.0% of the total factors and is primarily composed of PFBA and 6:2 FTS. As fluorinated surfactants are major constituents in AFFFs, firefighting training that utilizes AFFFs for fire control and suppression results in long-term contamination of groundwater and soil by environmental degradation products such as PFSAs and fluorinated thiosulfonic acids (FTSAs). As a result, PFBA and 6:2 FTS, which are short-chain substitutes for PFOS, were detected at elevated concentrations [74,82,83,84]. Factor 1 is therefore primarily attributed to wastewater discharges from gas stations. River water factor 2 represents 17.8% of the total factor loadings and is predominantly composed of PFDA. PFDA is widely present in domestic wastewater from detergents, food packaging, and other sources [85]. Due to low removal rates in conventional treatment processes, PFDA is also detected in the effluent from WWTPs, with some PFDA generated through the degradation of precursor compounds during treatment [86,87]. As shown in Figure 3, PFDA concentrations were low (all < 0.3 ng/L) and predominantly present near hospitals and sampling points with high human activity. Observations during sampling indicated that villagers washed clothes near these sites. Therefore, domestic wastewater is considered the primary source of factor 2. River factor 3 accounts for 19.0% of the total factor and is primarily composed of PFNA, with significant contributions from PFBA. Both PFBA and PFNA are extensively used as fluorinated surfactants, fabric finishing agents, and water- or oil-repellent coatings in the textile dyeing and electroplating industries [88,89]. Figure 3 indicates elevated PFBA and PFNA concentrations near Lingjian Creek, which is adjacent to industrial effluent discharge points and sampling sites with high human activity. Industrial effluent is therefore inferred as the potential source of river factor 3. River factor 4 accounts for 19.3% of the total variance and is primarily composed of PFHxS, with a partial contribution from PFHxA. Sewage treatment plants exhibit low removal rates for PFHxS and PFHxA, resulting in their enrichment in the effluent [90]. River factor 4 is thus inferred to originate from sewage treatment plants. River water factor 5 accounts for 23.9% of the total factor and is primarily composed of PFHxA and 6:2 FTS. PFHxA was detected at relatively high concentrations in irrigation water, and the minor presence of 6:2 FTS may originate from agricultural machinery lubricant leaks or soil residues [91]. The source of this factor is therefore agricultural wastewater.

Groundwater factor 1 accounts for 28.6% of the total factor, primarily composed of PFNA and PFPeA, with PFBA also contributing significantly. As shown in Figure 3, PFNA exhibited low detection rates and concentrations at the groundwater sampling sites. Its source may be wastewater discharge from treatment plants migrating into groundwater via rivers, warranting further investigation. Groundwater factor 2 accounts for 18.3% of the total variance and is primarily composed of PFHxS (75.4%) and 6:2 FTS (22.02%). PFHxS was detected at generally low concentrations in groundwater, with concentrations near villages and farmlands suggesting domestic wastewater as a potential source. Groundwater factor 3 accounted for 14.2% of the total variance, primarily composed of PFHxA. As shown in Figure 3, PFHxA exhibited higher concentrations at sampling point G10. The PFAS concentrations at G10 were significantly higher than those at sampling point Y29. This area features a scenic spot with high foot traffic and small-scale industries. Fluorochemical industrial parks represent a significant source of PFHxA, with the textile, semiconductor, and electroplating industries being major industrial contributors [92]. Factor 3 is presumed to originate from industrial wastewater discharges. Groundwater factor 4 accounts for 22.5% of the total factor loadings and is primarily composed of 6:2 FTS and PFPeA. An exceptionally high concentration of 6:2 FTS (73.6 ng/L) was detected at sampling point G8, where the PFAS concentrations also showed significant correlation with those in the river water at sampling point M9. Both sampling points are located near gas stations, and on-site investigations revealed that these gas stations conduct regular fire drills throughout the year. Groundwater factor 4 is therefore attributed to leakage or firefighting wastewater discharge from gas stations. These results confirm that wastewater from gas stations is the primary source of the 6:2 FTS anomaly in the groundwater of these karst regions. Groundwater factor 5 accounted for 16.4% of the total factor loadings, primarily composed of PFBA, with PFHxS also contributing. Statistics indicate that short-chain PFASs have been detected in fish across multiple regions nationwide [93]. Within the study area, PFBA exhibited the highest detected concentration at sampling point G5 near fishponds, suggesting aquaculture wastewater as a potential source. Given the Lijiang River basin’s extensive coverage and complex pollution dynamics from numerous tributaries, further investigation into pollution sources near the relevant sampling points is warranted.

By averaging the factor contributions from the river water and groundwater PMF models, the overall pollution source profile of PFASs in the Lijiang River basin was estimated. The results indicate that the main sources in the study area are sewage treatment plants (24.0%), gas station leaks/wastewater discharges (21.3%), agricultural/aquaculture wastewater (20.2%), untreated domestic sewage (18.1%), and small-scale industrial wastewater (16.7%). This integrated view of both river water and groundwater highlights that wastewater from sewage treatment plants and gas stations constitutes the dominant PFAS inputs, while agricultural and domestic sources also contribute substantially.

### 3.5. Ecological Risk Assessment

An ecological risk assessment was conducted for nine PFASs with high detection rates in the river water. Figure S4a presents the calculated ecological risks of each PFAS for algae, daphnia, and fish in these environments. The *RQ* values for the nine PFASs ranged from 6.45 × 10^−7^ to 2.85 × 10^−2^. PFUnDA posed a low risk to algae and no potential risk to daphnia or fish. The remaining PFASs did not present significant risks to any of the three aquatic organisms. Among the compounds assessed, PFUnDA (C11), PFDA (C10), and PFNA (C9) exhibited relatively higher *RQ* values for algae, daphnia, and fish, whereas PFBA (C4), PFPeA (C5), and PFBS (C4) demonstrated lower RQ values across all three taxa. These findings suggest that long-chain PFASs, due to their environmental persistence, high bioaccumulation potential, and toxicity, present more pronounced ecological risks and biological hazards [94,95]. PFAS contamination in the Lijiang River basin was lower than that in many other regions [60] and does not currently pose significant biological hazards. Nevertheless, the complex hydrological conditions associated with karst topography may facilitate PFAS accumulation in localized areas. Therefore, PFAS pollution in the Lijiang River basin continues to present potential risks, underscoring the need for enhanced monitoring and management.

### 3.6. Human Health Risk Assessment

This health risk assessment evaluated six frequently detected PFAS compounds in the groundwater (see Figure S4b). 6:2 FTS was excluded from the assessment because its elevated concentration (73.6 ng/L) was observed only at a single sampling point (G8) and does not represent the general exposure conditions across the study area. The *HQ* values ranged from 1.19 × 10^−3^ to 5.16 × 10^−2^ for all demographic groups, which were consistently below the 0.2 threshold. The highest *HQ* value (5.16 × 10^−2^) occurred in females aged 3–6 years for PFNA. Results indicate that current PFAS concentrations in the Lijiang River groundwater pose minimal direct health risks. While existing contamination levels across China demonstrate negligible health impacts, potential long-term ecological risks and cumulative bioeffects warrant consideration. Continuous environmental monitoring and rigorous assessment of PFAS distribution patterns remain essential, necessitating the implementation of preventive and control measures to safeguard public health and ecological integrity.

## 4. Conclusions

This study systematically investigated PFAS concentration levels, spatial distribution patterns, pollution sources, and ecological health risks within karst regions. Monitoring was conducted across contaminated sites, river water, and groundwater to characterize the distribution of PFASs in karst terrain. A parallel analysis employed PMF receptor modeling to trace contaminant pathways from sources to receiving water bodies and groundwater, integrating field investigation data with the PMF analytical results.

The study results demonstrate comparatively low PFAS concentrations in the Lijiang River, with ∑PFAS ranging from 0.08 to 74.0 ng/L. PFBA, PFHxA, PFNA, and 6:2 FTS were ubiquitously detected across all water matrices. Spatial distribution patterns correlated with WWTPs, anthropogenic activities, and domestic discharges, exhibiting lower PFAS concentrations in the main channel than in the tributaries. The predominance of short-chain PFASs over long-chain congeners aligns with global trends, reflecting regulatory-driven transitions toward novel substitutes. The short-chain alternative 6:2 FTS reached its peak concentration at G8 (73.6 ng/L), which was primarily attributable to firefighting wastewater from fuel service facilities. The distinctive hydrogeological characteristics of karst regions facilitate the transport of pollutants from point sources through fractures to the groundwater, resulting in greater contamination of groundwater. Water quality parameters (notably DO and pH) exhibited significant correlations with PFAS occurrences. While these findings are consistent with mechanistic studies, the complexity of aquatic ecosystems necessitates further investigation into the interactions between environmental parameters and PFASs. The ecological risk assessment indicated negligible risks to algae, daphnia, and fish for all PFASs except PFUnDA, which posed a low risk to algae. Groundwater PFAS exposure presents no immediate human health hazards.

Based on the distribution patterns of PFASs and the source apportionment results from the PMF model, PFAS contamination in this region primarily originates from domestic sewage, gas station leaks, and firefighting wastewater. The study reveals that PFAS contamination levels in groundwater are significantly higher than those in river water, which is primarily attributed to the unique geological structure of karst regions influencing pollutant migration and transformation. This phenomenon underscores the urgent need for enhanced dynamic monitoring and regulation of PFAS contamination in groundwater within karst areas.

## Figures and Tables

**Table 1:** Existence level of target PFAS in effluent, river water and groundwater.

Time	Water Bodies	Number of PFAS Species (Concentration Range/ng/L)	References
2020–2021	Water pollution source	17, (0.40–51.9)	[30]
2020	Water pollution source	73, (0.50–1140)	[31]
2020	Water pollution source	33, (0–31.4)	[32]
——	Water pollution source	10, (0.2–19,500.0)	[33]
2023	Water pollution source	4, (9400–18,000)	[34]
2019–2023	Water pollution source	10, (0–114)	[35]
2019–2023	Water pollution source	46, (0–12.7)	[36]
2020–2023	Water pollution source	32, (0–2420)	[37]
2021–2023	Water pollution source	28, (0–53.2)	[38]
2007–2019	Surface water	12, (1.06–13.7)	[19]
2019	Surface water	12, (0–72.0)	[39]
2019	Surface water	5, (0–59.0)	[40]
2021	Surface water	24, (0.24–2700)	[41]
2022	Surface water	12, (0~13,400)	[42]
2022–2023	Surface water	13, (0~134)	[43]
2023	Surface water	13, (0~16.4)	[44]
2023–2024	Surface water	12, (0.17–51.0)	[45]
2016–2022	Groundwater	38, (0–5,180,000)	[46]
2020	Groundwater	17, (0–1210)	[47]
2021	Groundwater	16, (0–8.09)	[48]
2021	Drinking Water	6, (0–22.0)	[49]
2023	Groundwater	8, (0–125)	[50]

## Data Availability

The Lijiang River PFAS data that support the findings of this study are available from Guilin University of Technology (GLUT), but restrictions apply to the availability of these data, which were used under license for the current study and so are not publicly available. The data are available upon request and with the permission of GLUT.

## Supplementary Materials

The following supporting information can be downloaded at: https://www.mdpi.com/article/10.3390/toxics14070548/s1. Text S1: Sampling procedure; Text S2: Sample pre-treatment steps; Text S3: UPLC-MS/MS experimental parameters; Table S1: Longitude and latitude information on river water sampling points; Table S2: Basic information of PFASs; Table S3: PFAS Chain length classification and distribution properties of PFASs; Table S4: Main chemical reagents; Table S5: Experimental main equipment and materials; Table S6: Gradient elution procedure for PFAS; Table S7: Mass spectrometry detection conditions for PFAS compounds; Table S8: Correlation coefficient (*R*^2^), limit of detection (*LOD*) and limit of quantitation (LOQ) of 24 PFASs; Table S9: Acute toxicity data *LC*_50_ (*EC*_50_) values for target PFAS to different aquatic organisms (mg/L) [22]; Table S10: Average daily acceptable amounts (ADIs) for different PFASs in humans; Table S11: Mean body weight (*BW*) and daily water intake (*DWI*) of our population in different age/sex groups; Table S12: *DWELs* (µg/L) for selected PFASs in groundwater for different age groups and genders; Table S13: HQ values (µg/L) for selected PFASs in groundwater for different age groups and genders; Table S14: PFAS concentrations (ng/L) in effluents, river water and groundwater; Table S15: Concentrations and detection rates of PFAS detected in effluent; Table S16: Concentrations and detection rates of PFAS detected in river water; Table S17: Concentrations and detection rates of PFAS detected in groundwater; Table S18: Effluent quality indicator detection results; Table S19: River water quality indicator detection results; Table S20: Groundwater quality indicator detection results; Table S21: RDA analysis results; Table S22: PMF analysis results of river water samplings (concentration of species); Table S23: PMF analysis results of river water samplings (percentage of species); Table S24: PMF analysis results of groundwater samplings (concentration of species); Table S25: PMF analysis results of groundwater samplings (percentage of species); Figure S1: Heat map of pearson correlation between PFAS and water quality parameters in effluents; Figure S2: Heat map of pearson correlation between PFAS and water quality parameters in river water; Figure S3: Heat map of Pearson correlation between PFAS and water quality parameters in groundwater; Figure S4: (a) Entropy of risk to algae, daphnia and fish for each PFAS in river water; (b) Health risk of each PFAS in groundwater for different age groups and genders.

## Abbreviations

The following abbreviations are used in this manuscript:

6:2 FTS	6:2 fluorotelomer sulfonate
AB	gastrointestinal absorption rate
ADI	acceptable daily intake
AFFF	aqueous film-forming foam
BW	body weight
CODMn	chemical oxygen demand determined by permanganate method (permanganate index)
DL	detection limit
DO	dissolved oxygen
DWEL	drinking water equivalent level
DWI	drinking water intake
EC	electrical conductivity
EC_50_	median effective concentration
FOE	exposure frequency
FTSAs	fluorinated thiosulfonic acids
GLUT	Guilin University of Technology
HQ	hazard quotient
HQ_mix_	cumulative health risk quotient
K_oc_	sediment-water partition coefficient
LC_50_	median lethal concentration
LOD	limit of detection
LOQ	limit of quantification
MEC	measured environmental concentration
NOEC	no-observed-effect concentration
OECD	Organization for Economic Cooperation and Development
PFAS	per- and polyfluoroalkyl substances
PFBA	perfluorobutanoic acid
PFBS	perfluorobutanesulfonate
PFCA	perfluorocarboxylic acid
PFDA	perfluorodecanoic acid
PFHpA	perfluoroheptanoic acid
PFHxA	perfluorohexanoic acid
PFHxS	perfluorohexanesulfonate
PFNA	perfluorononanoic acid
PFOA	perfluorooctanoic acid
PFPeA	perfluoropentanoic acid
PFPeS	perfluoropentanesulfonate
PFSA	perfluoroalkyl sulfonic acid
PFUnDA	perfluoroundecanoic acid
PMF	positive matrix factorization
PNEC	predicted no-effect concentration (or predicted non-effect concentration)
QL	quantification limit
*R*^2^	coefficient of determination (*R*-squared)
RDA	redundancy analysis
RQ	risk quotient
S/N	signal-to-noise ratio
TDS	total dissolved solids
TN	total nitrogen
TOC	total organic carbon
TP	total phosphorus
UPLC-MS/MS	ultra performance liquid chromatography–mass spectrometry/mass spectrometry
WWTP	wastewater treatment plant
