# Supplementary material for: Occurrence, Source Inference, and Risk Assessment of Per- and Polyfluoroalkyl Substances in Effluents, River Water and Groundwater from the Lijiang River Basin, a Typical Karst Region
Source: Toxics. 2026 Jun 24;14(7):548. doi: 10.3390/toxics14070548 (PMC13416516; doi:10.3390/toxics14070548)
Supplement: Supplementary file 1 [file toxics-14-00548-s001.zip › toxics-4313373-supplementary.pdf]

Supplementary Information

Occurrence, Source Inference, and Risk Assessment of Per- and Polyfluoroalkyl Substances in Effluents, River Water and Groundwater from the Lijiang River Basin, a Typical Karst Region

Supporting information contains:

Number of pages: 39

Text: S1-S3

Tables: S1-S25

Figures: S1-S4

Text S1. Sampling procedure

A portable multi-functional water quality analyzer was used to measure routine water quality parameters at each sampling point, including temperature, pH, dissolved oxygen (DO), oxidation-reduction potential (ORP), electrical conductivity (EC), and total dissolved solids (TDS). Water samples for routine parameters (1 L) and PFAS samples (500 mL) were collected in brown sampling bottles, with 0.5 mL of nitric acid added on-site. Samples were maintained at a low temperature and transported to the laboratory within 24 hours of collection. Upon returning to the laboratory, the samples were analyzed for the permanganate index (CODMn), chemical oxygen demand (COD), five-day biochemical oxygen demand (BOD_5), ammonia nitrogen (NH_4^+-N), nitrate nitrogen (NO_3^--N), nitrite nitrogen (NO_2^--N), total phosphorus (TP), and total nitrogen (TN). The JH-TD 401 series multi-parameter water quality analyzer was used to determine the NH_4^+-N, NO_3^--N, and NO_2^--N in the water samples.

Text S2. Sample pre-treatment steps

A 500 mL volume of the water sample was filtered through a glass fiber membrane filter. The polymeric weak anion exchange (PWAX) SPE column was activated using 4 mL of methanol containing 0.1% ammonia solution, 4 mL of methanol, and 4 mL of ultrapure water. The water sample was then loaded at a flow rate of 3 mL/min. The SPE column was eluted with 4 mL of 25 mM ammonium acetate buffer (pH = 4.0). The target compounds were eluted using 4 mL methanol and 4 mL methanol containing 0.1% ammonia solution. The eluate was collected in a 10 mL plastic centrifuge tube. Finally, the eluate was evaporated to near dryness under nitrogen, redissolved in 1 mL of 80% aqueous methanol solution, vortexed to homogenize, transferred to a 2 mL brown sample vial, and stored at −20 °C for subsequent instrument analysis. Before analysis, the sample was passed through a membrane filter and vortexed to homogenize.

Text S3. UPLC-MS/MS experimental parameters

An ACQUITY UPLC BEH C18 column (2.1 × 100 mm, 1.7 μm) was maintained at 35°C with a flow rate of 0.2 mL/min and a 10 μL injection volume. Mobile phase A comprised ultrapure water with 0.1% formic acid and 1 mmol ammonium acetate, while mobile phase B was acetonitrile. Mass spectrometry detection was performed using negative electrospray ionization (ESI^-) mode with multiple reaction monitoring (MRM). The capillary voltage was set at 3.5 kV, the ion source temperature at 150 °C, the desolvation temperature at 350 °C, the desolvation gas flow rate at 650 L/hr, and the cone voltage at 20 V.

Table S1. Longitude and latitude information on river water sampling points.

Sampling point type	Sample code	Sampling sites	Longitude	Latitude
Effluents	E1	Seventy-six Veterans Farm	110.386752	25.377018
	E2	Downstream of Beichong WWTP	110.275855	25.310233
	E3	Jiangan Road (Domestic Sewage)	110.328914	25.301547
	E4	Near Community Hospitals (Domestic Sewage)	110.290031	25.24923
	E5	Downstream of Qilidian WWTP	110.303611	25.240556
	E6	Downstream of Wayao WWTP	110.321324	25.220286
	E7	Fishpond	110.285986	25.166429
	E8	Near Farms	110.260643	25.15338
	E9	Downstream of Yanshan WWTP	110.297646	25.092845
Mainstem	M1	Guanjiang Bridge	110.365483	25.401508
	M2	Lingchuan County Drinking Water Source Protection Zone	110.310402	25.425145
	M3	Gantang River	110.327109	25.413762
	M4	Chengbei Waterworks (WW)	110.32191	25.352629
Mainstem	M5	Nanzhou Bridge	110.318056	25.320556
	M6	Yushan Bridge (Near Dongzhen Road WW)	110.301884	25.300814

Sampling point type	Sample code	Sampling sites	Longitude	Latitude
Mainstem	M7	Wayao WW	110.293773	25.24227
	M8	Longmen Bridge	110.341667	25.218889
	M9	Daxu Town, Fuli Village, Lower Li River	110.430987	25.129084
	M10	Yangshuo Li River, Guangxi	110.500128	24.790938
Taohuajiang River	T11	Jiashan Road	110.259694	25.29024
	T12	Rujia village, Lower Li River	110.269454	25.298469
	T13	Ludi Road	110.270746	25.304176
	T14	Xianren Bridge	110.275855	25.310233
	T15	Guanyi Bridge	110.284657	25.283918
Lingjianxi River	L16	Roadside	110.329905	25.288565
	L17	Huan Cheng Bei Road Bridge	110.31794	25.285886
	L18	Garden Bridge	110.315249	25.281578
	L19	Aroma Road Bridge	110.314145	25.277699
Xiaodongjiang River	X20	Longyin Bridge	110.300636	25.272867
	X21	Piercing Bridge	110.297202	25.263049
Nanxihe River	N22	South Creek Hill Bridge	110.279569	25.251515

Sampling point type	Sample code	Sampling sites	Longitude	Latitude
Huangshahe River	H23	Huangshahe River	110.388529	25.204925
Chaotianhe River	C24	Chaotianhe River	110.438	25.181497
Qifenghe River	Q25	Yujia Village	110.336485	25.191493
	Q26	Downstream of Grape Island	110.314837	25.159896
	Q27	Under the village of Xinqiaoyuan	110.297102	25.100169
Yulonghe River	Y28	Yulongbao Village	110.387581	24.826872
	Y29	Jinlong Bridge	110.389051	24.818902
	Y30	Old county town	110.43149	24.776252
Jinbaohe River	J31	Longtan Village	110.448193	24.729734
Groundwater	G1	Chiaki village well water	110.424103	25.501857
	G2	Sanjie village well water	110.385051	25.470591
	G3	Lujiang mountain spring Water	110.385051	25.470591
	G4	Ditang village well water	110.442549	25.403117
	G5	Kilotou village well water	110.313556	25.226492
	G6	Browser village well water	110.390406	25.20511
	G7	Qinyan village well water	110.434951	25.179072

Sampling point type	Sample code	Sampling sites	Longitude	Latitude
Groundwater	G8	Pok Lai village well water	110.431393	25.129336
	G9	Xinqiaoyuan village well water	110.298056	25.100967
	G10	Yulongbao village well water	110.389421	24.821182
	G11	Old county village well water	110.434951	24.775152
	G12	Longtan village well water	110.449154	24.730064
	G13	Kamiyama village well water	110.484748	24.791878

Table S2. Basic information of PFASs.

Types	Compound	Abbreviations	CAS	Molecular formula	Molecular mass
PFCAs[a]	Perfluorobutanoic acid	PFBA	375-22-4	$C_4HF_7O_2$	214.04
	Perfluoropentanoic acid	PFPeA	2706-90-3	$C_5HF_9O_2$	264.05
	Perfluorohexanoic acid	PFHxA	307-24-4	$C_6HF_{11}O_2$	314.05
	Perfluoroheptanoic acid	PFHpA	375-85-9	$C_7HF_{13}O_2$	364.04
	Perfluorooctanoic acid	PFOA	335-67-1	$C_8HF_{15}O_2$	414.07
PFCAs[a]	Perfluorononanoic acid	PFNA	375-95-1	$C_9HF_{17}O_2$	464.08
	Perfluorodecanoic acid	PFDA	335-76-2	$C_{10}HF_{19}O_2$	514.08
	Perfluoroundecanoic acid	PFUnDA	2058-94-8	$C_{11}HF_{21}O_2$	564.09

Types	Compound	Abbreviations	CAS	Molecular formula	Molecular mass
PFCAs[a]	Perfluorododecanoic acid	PFDoDA	307-55-1	$C_{12}HF_{23}O_2$	614.10
	Perfluorotridecanoic acid	PFTrDA	72629-94-8	$C_{13}HF_{25}O_2$	664.11
	Perfluorotetradecanoic acid	PFTeDA	376-06-7	$C_{14}HF_{27}O_2$	714.11
PFSAs[b]	Perfluorobutane sulfonate	PFBS	375-73-5	$C_4HF_9O_3S$	300.10
	Perfluoropentanesulfonate	PFPeS	2706-91-4	$C_5HF_{11}O_3S$	350.11
	Perfluorohexanesulfonate	PFHxS	355-46-4	$C_6HF_{13}O_3S$	400.11
	Perfluoroheptanesulfonate	PFHpS	375-92-8	$C_7HF_{15}O_3S$	450.12
	Perfluorooctanesulfonate	PFOS	1763-23-1	$C_8HF_{17}O_3S$	500.13
PFSAs[b]	Perfluorononanesulfonate	PFNS	98789-57-2	$C_9H_2F_{19}NaO_3S$	574.13
	Perfluorodecanesulfonate	PFDS	335-77-3	$C_{10}HF_{21}O_3S$	600.14
Others	Perfluorooctanesulfonamide	FOSA	754-91-6	$C_8H_2F_{17}NO_2S$	499.14
	N-methylperfluoro-1-octanesulfonamidoacetic acid	NMeFOSAA	2355-31-9	$C_{11}H_6F_{17}NO_4S$	571.21
	N-ethylperfluoro-1-octanesulfonamidoacetic acid	NEtFOSAA	2991-50-6	$C_{12}H_8F_{17}NO_4S$	585.23
	4:2 Fluorotelomer Sulfonate	4:2 FTS	757124-72-4	$C_6H_5F_9O_3S$	328.15
	6:2 Fluorotelomer Sulfonate	6:2 FTS	27619-97-2	$C_8H_5F_{13}O_3S$	428.19
	8:2 Fluorotelomer Sulfonate	8:2 FTS	39108-34-4	$C_{10}H_5F_{17}O_3S$	528.18

[a] Perfluorinated carboxylic acid, PFCAs.

[b] Perfluoroalkane sulfonic acids, PFSAs.

Table S3. PFAS Chain length classification and distribution properties of PFASs.

**Abbreviations**	**Chain length status[c]**	**Log K_{ow}**	**Water-soluble (25°C，mg/L)**	**Henry constant (25°C, atm-m^3/mol)**
PFBA	Short-chain	2.14	1373	1.19E-04
PFPeA	Short-chain	2.81	196.5	6.26E-04
PFHxA	Long-chain	3.48	27.12	3.29E^{-}3
PFHpA	Short-chain	4.15	3.647	1.16E-05
PFOA	Long-chain	4.81	0.4813	9.08E-02
PFNA	Long-chain	5.48	0.06258	4.77E-01
PFDA	Long-chain	6.15	0.008043	2.50E+00
PFUnDA	Long-chain	6.82	0.001024	1.32E+01
PFDoDA	Long-chain	7.49	0.0001293	6.93E+01
PFTrDA	Long-chain	8.16	1.62E-05	3.64E+02
PFTeDA	Long-chain	8.83	2.02E-06	1.91E+03
PFBS	Short-chain	1.82	344	1.44E-05

Abbreviations	Chain length status[a]	Log K_{ow}	Water-soluble (25°C，mg/L)	Henry constant (25°C, atm-m^3/mol)
PFPeS	Short-chain	2.49	46.56	7.56E-05
PFHxS	Long-chain	3.16	6.174	3.97E-04
PFHpS	Long-chain	3.82	0.8058	2.09E-03
PFOS	Long-chain	4.49	0.1039	1.10E-02
PFNS	Long-chain	5.16	0.01326	5.77E-02
PFDS	Long-chain	5.83	0.001678	3.03E-01
FOSA	Long-chain	5.80	0.008049	1.84E+00
NMeFOSAA	Long-chain	—	2.3×10-3	2.5×10-7
NEtFOSAA	Long-chain	6.22	0.002411	1.75E-03
4:2 FTS	Short-chain	—	0.15	N.A.[b]
6:2 FTS	Short-chain	2.66	10.97	7.00E-04
8:2 FTS	Long-chain	4.00	0.1821	1.93E-02

[a] Chain length status: Organization for Economic Cooperation and Development (OECD) regulations classify PFAS with six or more fully fluorinated carbon atoms as long-chain PFAS. Despite containing six fully fluorinated carbon atoms, 6:2 FTS is frequently considered a novel short-chain alternative.

[b] N.A.: indicates missing or unavailable data.

Table S4. Main chemical reagents.

Name of experimental reagents/materials	Purity/Specification	Manufacturer
disodium EDTA（Na_2EDTA）	Analytical Reagent	XiLONG SCIENTIFIC
concentrated hydrochloric acid（HCl）	Analytical Reagent	XiLONG SCIENTIFIC
caustic soda（NaOH）	Analytical Reagent	XiLONG SCIENTIFIC
acetonitrile	LC-MS	ANPEL Laboratory Technologies (Shanghai) Inc.
methanol	LC-MS	ANPEL Laboratory Technologies (Shanghai) Inc.
carboxylic acid	LC-MS	Shanghai Aladdin Biochemical Technology Co., Ltd.
deionized water	LC-MS	Merck and Co., Inc.

Table S5. Experimental main equipment and materials.

Instrument Name	Model Specification	Manufacturer
Ultra performance liquid chromatography triple quadrupole tandem mass spectrometer	ACQUITYUPLC-I-Class/XevoTQ-Smicro	Waters, USA
12-well vacuum solid phase extraction device	5982-9110Agilent	AgilentTechnologiesInc, USA
Ceramic fiber muffle furnace	SX3-1.5-10	Hangzhou Zhuochi Instrument Co., Ltd.
24-position Nitrogen Blow Concentrator	N-EVAP-24	Agilent Technologies Inc.

Instrument Name	Model Specification	Manufacturer
Benchtop circulating water type multi-purpose Vacuum pump	SHB-III	Zhengzhou Great Wall Scientific Industrial and Trade Co., Ltd.
Electronic analytical balance	ME203E	MettlerToledo, Switzerland
Vortex mixer	VortexGenie2	ScientificIndustries, USA
Freeze dryer	FD-1A-50	Beijing Boyikang Lab Instrument Co., Ltd.
Dual-frequency ultrasonic cleaner	KQ5200	Kun Shan Ultrasonic Instruments Co., Ltd.
Multi-parameter water quality analyzer	SMARTOLLTMMP	In-SituInc,USA
Ultrapure water meter	Milli-Q	Millipore China Ltd. Co.
pH meters	sartoius	Beijing Sartorius Instruments Inc.
Portable dissolved oxygen meter	JPBJ-608	INASE Scientific Instrument CO., LTD
Nylon 66 needle filter	0.22μm×13mm	Tianjin Jinteng Experimental Equipment Co.
GF/F glass fiber filter membrane	0.45μm	Whatman,England
Needle nylon membrane filter	0.22μm/13mm	Tianjin Jinteng Experimental Equipment Co.
HLB solid phase extraction columns	500mg/6mL	ANPEL Laboratory Technologies (Shanghai) Inc.
PWAX weak anion exchange SPE columns	150mg/6mL	ANPEL Laboratory Technologies (Shanghai) Inc.

Table S6. Gradient elution procedure for PFAS.

Time (min)	flow rates (mL/min)	A%	B%	curve
Initial	0.20	90	10	6
2.00	0.20	50	50	6
8.00	0.20	5	95	6
10.00	0.20	5	95	6
10.50	0.20	90	10	6
14.00	0.20	90	10	6

Table S7. Mass spectrometry detection conditions for PFAS compounds.

Compound	Peak departure time (min)	Parent ion (m/z)	daughter ion (m/z)	Cone hole voltage (V)	Collision energy (V)
PFBA	2.91	213	169.2	10	10
PFPeA	3.34	263	219.2	10	5
PFHxA	3.75	313	269.2	5	10
PFHpA	4.23	363	319.2	15	10
PFOA	4.79	413	369.2	10	10
PFNA	5.40	463	419.2	10	10
PFDA	6.07	513	469.2	15	10

Compound	Peak departure time (min)	Parent ion (m/z)	daughter ion (m/z)	Cone hole voltage (V)	Collision energy (V)
PFUnDA	6.76	563	519.2	25	10
PFDoDA	7.41	613	569.2	30	10
PFTrDA	8.05	663	619.2	5	10
PFTeDA	8.63	713	669.2	10	15
PFBS	3.89	299	80.2	15	30
PFPeS	4.39	349	80.2	10	30
PFHxS	4.92	399	80.2	10	35
PFHpS	5.52	449	80.2	15	35
PFOS	6.14	499	80.2	15	40
PFNS	6.77	549	80.2	20	40
PFDS	7.37	513	469.2	15	10
FOSA	6.91	498	78.2	40	30
NMeFOSAA	7.43	570	419.2	35	20
NEtFOSAA	7.79	584	419.2	15	20
4:2FTS	3.45	327	307.2	15	15

Compound	Peak departure time (min)	Parent ion (m/z)	daughter ion (m/z)	Cone hole voltage (V)	Collision energy (V)
6:2FTS	4.29	427	407.2	15	20
8:2FTS	5.45	527	507.2	15	25

Table S8. Correlation coefficient (R^2), limit of detection (LOD) and limit of quantitation (LOQ) of 24 PFASs.

Compound	Linear equation	Linear coefficient R^2	LOD (ng/L)	LOQ (ng/L)
PFBA	y=1180.65x-154.294	0.9955	0.06	0.20
PFPeA	y=1985.81x-180.154	0.9943	0.15	0.50
PFHxA	y=1590.69x-139.112	0.9943	0.06	0.20
PFHpA	y=1908.37x+797.598	0.996	0.67	2.23
PFOA	y=2541.26x+2099.63	0.9942	0.07	0.23
PFNA	y=2582.27x+130.828	0.9955	0.04	0.13
PFDA	y=2881.44x+1550.2	0.9939	0.01	0.03
PFUnDA	y=2678.87x+1261.07	0.9967	0.02	0.07
PFDoDA	y=3427.63x+1622.38	0.9942	0.01	0.03
PFTrDA	y=8212.66x-565.701	0.9946	0.33	1.10
PFTeDA	y=4935.27x+1125.75	0.9947	0.11	0.37

Compound	Linear equation	Linear coefficient R^2	LOD (ng/L)	LOQ (ng/L)
PFBS	y=662.064x+527.521	0.9957	0.02	0.07
PFPeS	y=577.561x+489.429	0.9963	0.19	0.63
PFHxS	y=623.884x-18.4878	0.9953	0.01	0.03
PFHpS	y=598.095x+832.523	0.9938	0.51	1.70
PFOS	y=784.411x-263.577	0.9932	0.02	0.07
PFNS	y=646.537x+487.013	0.9937	0.02	0.07
PFDS	y=585.999x+613.714	0.9945	0.03	0.10
FOSA	y=1626.02x+1059.71	0.9937	0.01	0.03
NMeFOSAA	y=402.796x-125.238	0.9962	0.01	0.03
NEtFOSAA	y=558.734x+273.872	0.9938	0.04	0.13
4:2 FTS	y=291.233x+51.6162	0.9952	0.95	3.17
6:2 FTS	y=332.212x+486.687	0.9942	0.13	0.43
8:2 FTS	y=279.932x+12.3105	0.9942	0.03	0.10

Table S9. Acute toxicity data LC_{50} (EC_{50}) values for target PFAS to different aquatic organisms (mg/L).

Compound	MEC (ng/L)	LC_{50} (EC_{50}) (mg/L)			
		Algae	Daphnia	Fish	Data sources
PFBA	1.03	597	761	1320	ECOSAR

Compound	MEC (ng/L)	LC_{50} (EC_{50}) (mg/L)			
		Algae	Daphnia	Fish	Data sources
PFPeA	4.33	254	250	409	ECOSAR
PFHxA	2.81	42.3	79.3	122.0	ECOSAR
PFNA	1.60	0.26	2.22	6.26	ECOSAR
PFDA	0.21	0.05	0.66	2.39	ECOSAR
PFUnDA	0.28	0.01	0.19	0.90	ECOSAR
PFBS	2.45	1400	2010	3800	ECOSAR
PFHxS	0.66	130	190	301	ECOSAR
6:2 FTS	12.30	125	112	108	[1]

Table S10. Average daily acceptable amounts (ADIs) for different PFASs in humans.

Compound	ADI (μg/kg bw/day)
PFHxA	100
PFNA	4.15
PFDA	6
PFUnDA	5.05
PFBS	500

Compound	ADI (μg/kg bw/day)
PFHxS	5

Table S11. Mean body weight (BW) and daily water intake (DWI) of our population in different age/sex groups.

Age grouping	Genders	BW/kg	DWI (L/day)
3-6 years old	males	19.63	1.08
	females	18.65	1.08
7-11 years old	males	33.84	1.24
	females	31.94	1.24
12-16 years old	males	55.16	1.73
	females	49.44	1.73
17-19 years old	males	63.43	2.26
	females	52.67	2.26
20-24 years old	males	67.2	2.81
	females	53.8	2.81
25-59 years old	males	70.77	2.81
	females	58.37	2.81
>60 years old	males	67.1	2.81
	females	59.45	2.81

Table S12. DWELs (μg/L) for selected PFASs in groundwater for different age groups and genders.

Age grouping	Genders	DWELs (µg/L)					
		PFHxA	PFNA	PFDA	PFUnDA	PFBS	PFHxS
3-6 years old	males	378.7	15.7	22.7	19.1	1893.3	18.9
	females	359.8	14.9	21.6	18.2	1798.8	18.0
7-11 years old	males	568.5	23.6	34.1	28.7	2842.7	28.4
	females	536.6	22.3	32.2	27.1	2683.1	26.8
12-16 years old	males	664.3	27.6	39.9	33.5	3321.3	33.2
	females	595.4	24.7	35.7	30.1	2976.9	29.8
17-19 years old	males	584.7	24.3	35.1	29.5	2923.6	29.2
	females	485.5	20.1	29.1	24.5	2427.6	24.3
20-24 years old	males	498.2	20.7	29.9	25.2	2491.1	24.9
	females	398.9	16.6	23.9	20.1	1994.4	19.9
25-59 years old	males	524.7	21.8	31.5	26.5	2623.4	26.2
	females	432.8	18.0	26.0	21.9	2163.8	21.6
>60 years old	males	497.5	20.6	29.8	25.1	2487.4	24.9
	females	440.8	18.3	26.4	22.3	2203.8	22.0

Table S13. HQ values (µg/L) for selected PFASs in groundwater for different age groups and genders.

Age grouping	Genders	HQ Values (μg/L)						HQ_{mix}
		PFHxA	PFNA	PFDA	PFUnDA	PFBS	PFHxS	
3-6 years old	males	5.0E-03	4.9E-02	7.5E-03	2.1E-03	5.5E-03	1.0E-02	7.9E-02
	females	5.3E-03	5.2E-02	7.9E-03	2.2E-03	5.7E-03	1.1E-02	8.3E-02
7-11 years old	males	3.4E-03	3.3E-02	5.0E-03	1.4E-03	3.6E-03	6.7E-03	5.3E-02
	females	3.6E-03	3.5E-02	5.3E-03	1.5E-03	3.8E-03	7.1E-03	5.6E-02
12-16 years old	males	2.9E-03	2.8E-02	4.3E-03	1.2E-03	3.1E-03	5.7E-03	4.5E-02
	females	3.2E-03	3.1E-02	4.8E-03	1.3E-03	3.5E-03	6.4E-03	5.0E-02
17-19 years old	males	3.3E-03	3.2E-02	4.8E-03	1.4E-03	3.5E-03	6.5E-03	5.1E-02
	females	3.9E-03	3.8E-02	5.8E-03	1.6E-03	4.3E-03	7.8E-03	6.2E-02
20-24 years old	males	3.8E-03	3.7E-02	5.7E-03	1.6E-03	4.1E-03	7.6E-03	6.0E-02
	females	4.8E-03	4.7E-02	7.1E-03	2.0E-03	5.2E-03	9.5E-03	7.5E-02
25-59 years old	males	3.6E-03	3.5E-02	5.4E-03	1.5E-03	3.9E-03	7.2E-03	5.7E-02
	females	4.4E-03	4.3E-02	6.5E-03	1.8E-03	4.8E-03	8.8E-03	6.9E-02
>60 years old	males	3.8E-03	3.7E-02	5.7E-03	1.6E-03	4.1E-03	7.6E-03	6.0E-02
	females	4.3E-03	4.2E-02	6.4E-03	1.8E-03	4.7E-03	8.6E-03	6.8E-02

Table S14. PFAS concentrations (ng/L) in effluents, river water and groundwater.

PFAS	Effluents								River water		Groundwater	
	WWTPs		Hospitals		Farms		Fish pond					
	Range	Average	Range	Average	Range	Average	Range	Average	Range	Average	Range	Average
PFBA	<0.06-0.60	0.33±0.26	<0.06-0.32	0.16±0.23	0.25-0.33	0.29±0.05	0.71	0.71	<0.06-1.03	0.20±0.26	<0.06-3.41	0.60±0.98
PFPeA	<0.06-4.33	1.25±2.06	<0.06-0.25	0.13±0.18	<0.06-0.51	0.26±0.36	0.38	0.38	<0.06-1.01	0.23±0.33	<0.06-0.60	0.14±0.22
PFHxA	0.43-2.74	1.06±1.13	0.35-2.81	1.60±1.74	0.09-0.18	0.14±0.07	0.18	0.18	<0.06-0.90	0.37±0.38	<0.06-1.91	0.33±0.52
PFHpA	<0.67	——	<0.67	——	<0.67	——	<0.67	——	<0.67-3.26	0.14±0.61	<0.67	——
PFOA	<0.07	——	<0.07	——	<0.07	——	<0.07	——	<0.07	——	<0.07-0.50	0.05±0.14
PFNA	<0.04-1.48	0.64±0.69	<0.04-1.60	0.80±1.13	<0.04-0.10	0.05±0.07	0.97	0.97	<0.04-1.59	0.38±0.48	<0.04-0.77	0.14±0.22

PFAS	Effluents								River water		Groundwater	
	WWTPs		Hospitals		Farms		Fish pond					
	Range	Average	Range	Average	Range	Average	Range	Average	Range	Average	Range	Average
PFUnDA	<0.02	——	<0.02-0.28	0.14±0.20	<0.02	——	<0.02	——	<0.02-0.10	0.01±0.03	<0.02-0.04	0.01±0.02
PFBS	<0.02-0.41	0.10±0.21	0.21-0.76	0.49±0.39	<0.02	——	<0.02	——	<0.02-2.45	0.20±0.52	<0.02-10.32	0.80±2.86
PFPeS	<0.19	——	<0.19-1.68	0.84±1.19	<0.19	——	<0.19	——	<0.19	——	<0.19	——
PFHxS	<0.01-0.66	0.21±0.31	<0.01-0.09	0.05±0.06	<0.01	——	<0.01	——	<0.01-0.28	0.03±0.07	<0.01-0.19	0.04±0.07
6:2 FTS	<0.13-12.3	3.30±6.00	0.64-1.68	1.16±0.74	0.42-0.44	0.43±0.01	0.54	0.54	<0.13-10.8	0.72±1.92	<0.13-73.6	5.83±20.37
∑PFAS	1.96-21.9	6.89±10.0	4.50-6.31	5.41±1.28	0.78-1.55	1.17±0.55	2.78	2.78	0.08-11.7	2.31±2.40	0.08-74.0	7.97±20.22

Table S15. Concentrations and detection rates of PFAS detected in effluent.

Category	No. of carbon in the chain	Target analytes	Min[a] (ng/L)	Max[b] (ng/L)	Mean[c] (ng/L)	Med[d] (ng/L)	Freq[e] (%)
PFCAs	C4	PFBA	ND	0.71	0.33	0.32	77.8
	C5	PFPeA	ND	4.33	0.68	0.33	66.7
	C6	PFHxA	0.09	2.81	0.87	0.43	100
	C7	PFHpA	ND	0.14*	-	-	0
	C9	PFNA	ND	1.60	0.58	0.14	66.7
	C10	PFDA	ND	0.12	0.02	0	33.3
	C11	PFUnDA	ND	0.29	0.03	0	11.1
		∑PFCA	0.34	8.54	2.51	1.51	100
PFSAs	C4	PFBS	ND	0.77	0.15	0	33.3
	C5	PFPeS	ND	1.68	0.19	0	11.1
	C6	PFHxS	ND	0.66	0.10	0	33.3
		∑PFSA	ND	1.89	0.44	0	37.5
FOSA	C8	6:2 FTS	ND	12.3	1.88	0.52	88.9
		∑PFAS	0.78	21.9	4.83	2.03	100

[a] Minimum concentration.

[b] Maximum concentration.

[c] Mean value of PFASs concentrations.

[d] Median concentration.

[e] Frequency (%) of detection of each PFAS in all samples.

ND: Not Detected.

[*] PFAS concentration < LOD.

Table S16. Concentrations and detection rates of PFAS detected in river water.

Category	No. of carbon in the chain	Target analytes	Min[a] (ng/L)	Max[b] (ng/L)	Mean[c] (ng/L)	Med[d] (ng/L)	Freq[e] (%)
PFCAs	C4	PFBA	ND	1.03	0.20	0.11	61.3
	C5	PFPeA	ND	1.01	0.23	0	45.2
	C6	PFHxA	ND	1.90	0.37	0.34	77.4
	C7	PFHpA	ND	3.26	0.14	0	6.45
	C9	PFNA	ND	1.59	0.38	0.15	87.1
	C10	PFDA	ND	0.21	0.04	0.01	51.6
	C11	PFUnDA	ND	0.10	0.01	0	12.9
		∑PFCA	0.08	4.73	1.42	1.01	100
PFSAs	C4	PFBS	ND	2.45	0.20	0	19.4
PFSAs	C6	PFHxS	ND	0.28	0.03	0	22.6

Category	No. of carbon in the chain	Target analytes	Min[a] (ng/L)	Max[b] (ng/L)	Mean[c] (ng/L)	Med[d] (ng/L)	Freq[e] (%)
PFSAs	C6	∑PFSA	ND	2.45	0.23	0	35.5
FOSA	C8	6:2 FTS	ND	10.8	0.72	0.34	71
		∑PFAS	0.08	11.7	2.31	1.61	100

[a] Minimum concentration.

[b] Maximum concentration.

[c] Mean value of PFASs concentrations.

[d] Median concentration.

[e] Frequency (%) of detection of each PFASs in all samples.

ND: Not Detected.

Table S17. Concentrations and detection rates of PFAS detected in groundwater.

Compound	No. of carbon in the chain	Target analytes	Min[a] (ng/L)	Max[b] (ng/L)	Mean[c] (ng/L)	Med[d] (ng/L)	Freq[e] (%)
PFCAs	C4	PFBA	ND	3.41	0.60	0.12	76.9
	C5	PFPeA	ND	0.60	0.14	0	38.5
	C6	PFHxA	ND	1.91	0.33	0.18	76.9
	C8	PFOA	ND	0.50	0.05	0	15.4
PFCAs	C9	PFNA	ND	0.77	0.14	0.05	61.5

Compound	No. of carbon in the chain	Target analytes	Min[a] (ng/L)	Max[b] (ng/L)	Mean[c] (ng/L)	Med[d] (ng/L)	Freq[e] (%)
PFCAs	C10	PFDA	ND	0.17	0.03	0	38.5
	C11	PFUnDA	ND	0.04	0.01	0	30.8
		∑PFCA	0.08	5.26	1.29	0.69	100
PFSAs	C4	PFBS	ND	10.3	0.80	0	15.4
	C6	PFHxS	ND	0.19	0.04	0	30.8
		∑PFSA	ND	10.5	0.84	0	38.5
FOSA	C8	6:2 FTS	ND	73.6	5.83	0	46.2
		∑PFAS	0.08	74.0	7.97	0.90	100

[a] Minimum concentration.

[b] Maximum concentration.

[c] Mean value of PFASs concentrations.

[d] Median concentration.

[e] Frequency (%) of detection of each PFASs in all samples.

ND: Not Detected.

Table S18. Effluent quality indicator detection results.

Sample code	pH	EC	DO	ORP	TOC	COD	TDS	NO_2^--N	NH_4^+-N	NO_3^--N	TN	TP	CODMn
E1	7.90	0.22	9.44	248	3.60	3.50	85.0	0.01	0.04	1.00	28.0	0.07	1.86
E2	6.92	0.43	7.37	190	4.50	5.20	159	0.06	0.49	2.01	5.87	0.09	5.05
E3	6.95	1.03	1.41	185	20.1	18.0	434	0.04	13.5	0.34	76.8	2.22	9.86
E4	7.17	0.58	4.73	190	3.60	3.50	225	0.21	2.02	0.00	5.91	0.14	3.17
E5	7.23	0.27	9.03	206	3.40	3.20	107	0.03	0.30	0.31	3.02	0.04	2.45
E6	7.10	0.66	7.84	206	4.40	5.10	270	0.16	0.88	0.00	10.7	0.18	4.54
E7	7.37	0.33	9.26	161	20.1	20.0	134	0.07	1.23	0.77	8.94	0.32	5.06
E8	7.37	0.35	9.10	164	3.40	3.20	143	0.00	0.14	0.02	0.75	0.02	2.53
E9	7.38	0.52	8.78	161	3.40	3.20	211	0.03	0.23	0.91	3.64	0.01	1.24

Note: pH is dimensionless, electrical conductivity (EC) is in ms/cm, oxidation reduction potential (ORP) is in mv, total dissolved solids (TDS) is in ppm, and the rest of the water quality indexes are in mg/L

Note: The presence of low-molecular-weight organic compounds and reduced inorganic species in the karst waters of the Lijiang River basin may cause differences in oxidation selectivity between the dichromate method (COD) and the permanganate method (CODMn), resulting in occasionally observed TOC > COD or CODMn > COD values. All TOC data were obtained after rigorous inorganic carbon removal pretreatment and represent pure total organic carbon, not total carbon. Given these analytical limitations, the conventional water quality parameters in these tables (Tables S18–S20) are provided for auxiliary reference only and were not used as the primary basis for the core conclusions of this study.

Table S19. River water quality indicator detection results.

Sample code	pH	EC	DO	ORP	TOC	COD	TDS	NO_2^--N	NH_4^+-N	NO_3^--N	TN	TP	CODMn
M1	7.25	0.14	9.16	2280	2.7	2.4	53	0.002	0.023	0.68	31.7	0.102	1.01
M2	7.7	0.13	9.24	292	2.2	1.7	42	0.005	0.044	0.48	27.6	0.069	1.26
M3	8.72	0.13	9.59	293	2.4	2	50	0.005	0.132	0.11	30.6	0.682	1.94
M4	8.28	0.17	9.42	287	2.5	2.2	63	0.009	0.071	0.17	8.81	0.031	1.69
M5	7.23	0.19	9.24	330	2.2	1.8	72	0.011	0.05	0.28	18.8	0.028	1.37
M6	7.18	0.48	8.41	185	1.9	1.3	186	0.014	0.078	1.6	8.45	0	1.89
M7	7.3	0.23	9.25	189	2.6	2.3	91	0.02	0.234	0.2	5.47	0.021	1.93
M8	7.19	0.27	9.29	200	3.1	2.7	109	0.046	0.349	0	10.1	0.045	2.94
M9	7.04	0.29	9.4	173	2.9	2.6	117	0.022	0.037	1.95	2.62	0.043	1.34
M10	7.21	0.27	9.03	208	3.3	3	104	0.005	0.015	2.44	4.49	0.024	2.64
T11	6.85	0.47	6.89	180	4.8	6	191	0.148	0.465	1.89	5.96	0.083	4.69
T12	6.88	0.43	7.43	186	4.6	5.5	175	0.123	0.512	2.41	6.27	0.156	2.81
T13	6.91	0.43	8	187	4.5	5.2	167	0.095	0.444	2.84	7.21	0.061	3.47
T14	7.14	0.49	7.53	186	19.9	13.5	26	0.04	3.751	1.09	8.81	0.259	3.31
T15	7.34	0.21	7.43	195	2.6	2.4	80	0.055	0.397	2.24	3.15	0.043	2.08

Sample code	pH	EC	DO	ORP	TOC	COD	TDS	NO_2^--N	NH_4^+-N	NO_3^--N	TN	TP	CODMn
L16	6.89	0.58	4.81	362	4.6	5.5	239	0.143	5.068	0.63	7.34	0.199	2.55
L17	7.64	0.59	3.54	300	15.1	10.3	243	0.157	5.258	0	21.4	0.153	6.71
L18	7.66	0.57	5.4	326	10.6	7.2	238	0.132	5.516	1.32	10.2	0.193	3.03
L19	7.09	0.56	4.72	343	4.4	5.2	220	0.136	6.481	0	12.8	0.168	2.94
X20	7.33	0.19	8.23	197	2.4	2.1	71	0.014	0.295	0	3.02	0.014	1.62
X21	7.4	0.19	8.88	194	2.4	2.1	68	0.009	0.105	0	4.27	0.016	2.06
N22	7.2	0.57	6.14	188	2.6	2.3	88	0.164	4.199	0	13.2	0.229	2.25
H23	6.71	0.42	8.78	172	2.8	2.5	163	0.011	0.003	1.12	4.04	0.024	8.39
C24	6.95	0.27	8.92	169	3.1	2.7	107	0.011	0.03	1.78	15.1	0.03	0.78
Q25	7.09	0.52	8.53	190	4	4.3	216	0.105	0.268	0.54	5.78	0.139	2.81
Q26	7.04	0.56	7.32	175	3.7	3.6	233	0.116	0.37	2.29	8.01	0.176	1.68
Q27	7.46	0.6	8.4	174	3.8	3.8	247	0.202	0.39	3.01	11.1	2.216	2.44
Y28	6.93	0.49	8.27	188	2.5	2.2	204	0.003	0.023	3.21	5.56	0.013	3.81
Y29	7.26	0.39	7.64	192	2.1	1.6	148	0.011	0.023	2.03	3.51	0.021	3.05

Sample code	pH	EC	DO	ORP	TOC	COD	TDS	NO_2^--N	NH_4^+-N	NO_3^--N	TN	TP	CODMn
Y30	7.18	0.44	8.26	194	2.1	1.5	149	0.014	3.357	1.55	3.46	0.012	1.47
J31	7.14	0.36	5.23	198	2.4	2.1	129	0.008	0.675	1.46	4.36	0.059	2.18

Note: pH is dimensionless, electrical conductivity (EC) is in ms/cm, oxidation reduction potential (ORP) is in mv, total dissolved solids (TDS) is in ppm, and the rest of the water quality indexes are in mg/L.

Table S20. Groundwater quality indicator detection results.

Sample code	pH	EC	DO	ORP	TOC	COD	TDS	NO_2^--N	NH_4^+-N	NO_3^--N	TN	TP	CODMn
G1	6.82	0.49	6.65	251	2.2	1.8	196	0.006	0.091	4.79	21.6	0.034	0.85
G2	7.01	0.7	5.43	234	1.8	1.2	289	0.02	0.451	5.54	11.5	0.016	0.44
G3	5.99	0.01	8.65	303	1.2	0.6	3	0	0	0.31	23.6	0.026	8.63
G4	6.85	0.42	8.75	266	2.1	1.5	167	0.011	0.064	2.26	20.8	0.031	0.92
G5	7.04	0.58	6.76	208	1.9	1.3	241	0	0	6.2	16.7	0	1.96
G6	6.28	0.71	7.1	163	1.8	1.2	316	0	0	10.19	15.8	0.175	0.51
G7	6.57	0.53	8.52	169	1.6	0.9	208	0	0	6.89	0.35	0.021	0.88
G8	6.8	0.3	6.51	193	1.6	0.9	121	0	0	2.21	4.49	0.017	1.66
G9	7.28	0.8	4.96	188	1.9	1.3	321	0.131	0.01	1.55	4.89	0.026	1.83
G10	6.53	0.48	7.45	189	2.6	2.3	192	0	0.193	3.44	5.25	0.038	2.03

Sample code	pH	EC	DO	ORP	TOC	COD	TDS	NO_2^--N	NH_4^+-N	NO_3^--N	TN	TP	CODMn
G11	7.16	0.48	5.67	198	2.2	1.7	205	0	0.01	1.63	2.66	0.011	1.90
G12	7.25	0.24	8.34	194	3.3	3	93	0.006	7.648	1.32	1.59	0.021	2.11
G13	6.64	0.81	8.47	189	1.9	1.2	331	0	0.003	4.42	12.2	0.002	2.24

Note: pH is dimensionless, electrical conductivity (EC) is in ms/cm, oxidation reduction potential (ORP) is in mv, total dissolved solids (TDS) is in ppm, and the rest of the water quality indexes are in mg/L

Table S21. RDA analysis results.

Importance of components	RDA1	RDA2	RDA3	RDA4	RDA5
Eigenvalue	1.4232	0.8859	0.16619	0.11493	0.023604
Proportion explained	0.2846	0.1772	0.03324	0.02299	0.004721
Cumulative proportion	0.2846	0.4618	0.49505	0.51804	0.522758

Table S22. PMF analysis results of river water samplings (concentration of species).

Compound	Factor 1	Factor 2	Factor 3	Factor 4	Factor 5
PFBA	0.11744	0.0030597	0.075755	0	0
PFPeA	0	0.062723	0	0.046258	0.22196
PFHxA	0	0	0.28187	0.040774	0.064256
PFNA	0.0042948	0.033985	0.00089299	0.00021065	0

Compound	Factor 1	Factor 2	Factor 3	Factor 4	Factor 5
PFHxS	0.0023678	0	0	0.023884	0
6:2 FTS	0.16007	0	0	0	0.23772

Table S23. PMF analysis results of river water samplings (percentage of species).

Compound	Factor 1	Factor 2	Factor 3	Factor 4	Factor 5
PFBA	59.84060509	1.559045465	38.60034944	0	0
PFPeA	0	18.95292514	0	13.97771808	67.06935677
PFHxA	0	0	72.8534505	10.53864048	16.60790902
PFNA	10.90509107	86.29261436	2.26742509	0.534869478	0
PFHxS	9.019571991	0	0	90.98042801	0
6:2 FTS	40.23982503	0	0	0	59.76017497

Table S24. PMF analysis results of groundwater samplings (concentration of species).

Compound	Factor 1	Factor 2	Factor 3	Factor 4	Factor 5
PFBA	0.15656	0.015085	0	0.0035475	0.42758
PFPeA	0.094781	1.0962E-06	0.0081372	0.079997	0.0051744
PFHxA	3.8198E-06	0.0064597	0.16757	0.034777	0
PFNA	0.13627	0.010248	0.0005137	0	0.00016911

Compound	Factor 1	Factor 2	Factor 3	Factor 4	Factor 5
PFHxS	0	0.036021	1.2206E-07	5.1032E-08	0.011755
6:2 FTS	0.0060761	0.047753	0	0.16301	2.5949E-06

Table S25. PMF analysis results of groundwater samplings (percentage of species).

Compound	Factor 1	Factor 2	Factor 3	Factor 4	Factor 5
PFBA	25.97331497	2.502602557	0	0.588530499	70.93555197
PFPeA	50.39111552	0.000582804	4.326210793	42.53107762	2.751013264
PFHxA	0.001829314	3.093570193	80.24978826	16.65481224	0
PFNA	92.57421885	6.961918212	0.348979058	0	0.114883879
PFHxS	0	75.39532296	0.000255483	0.000106815	24.60431474
6:2 FTS	2.802090254	22.02205624	0	75.17465683	0.001196679

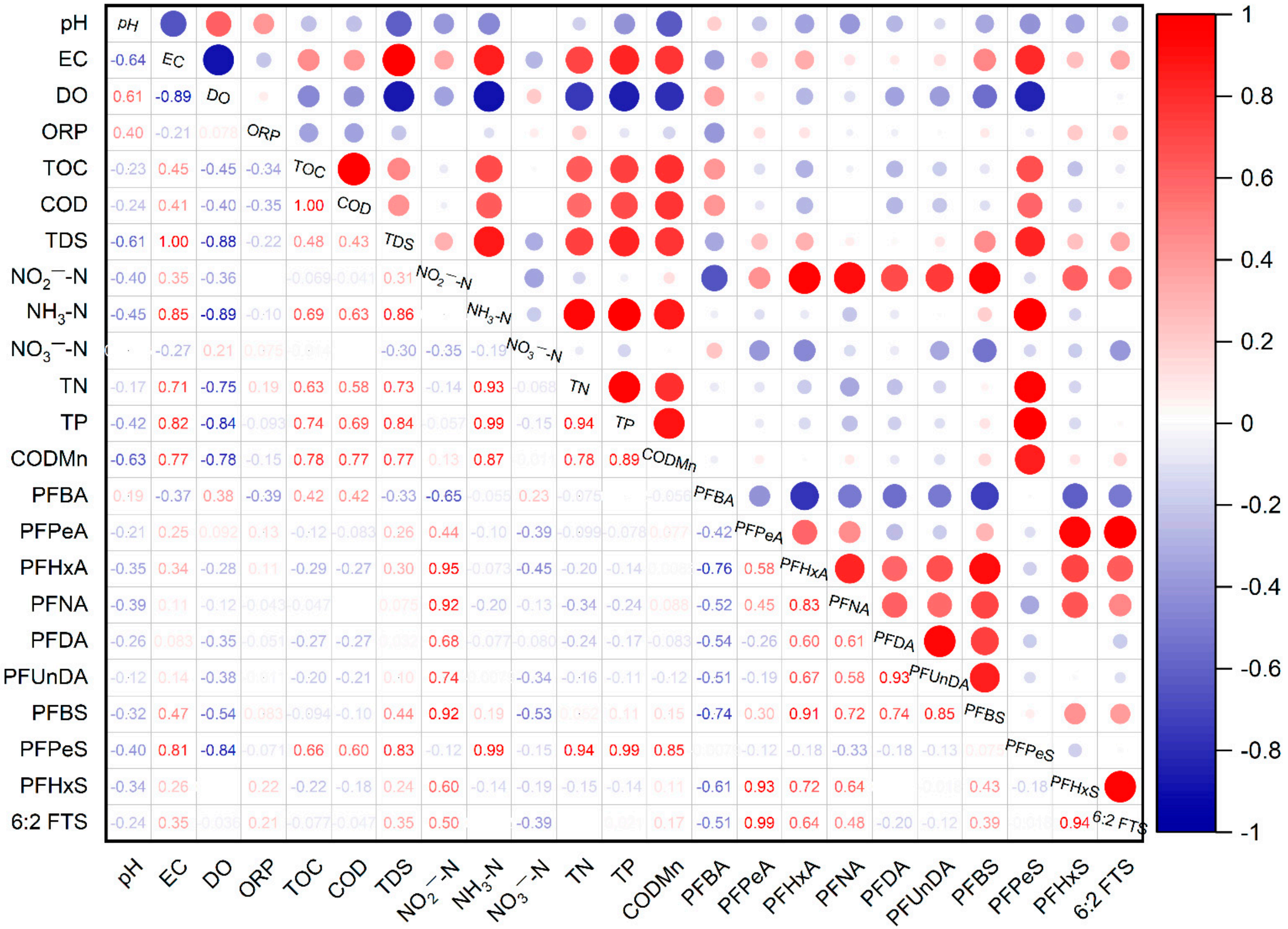

Figure S1 Heat map of pearson correlation between PFAS and water quality parameters in effluents

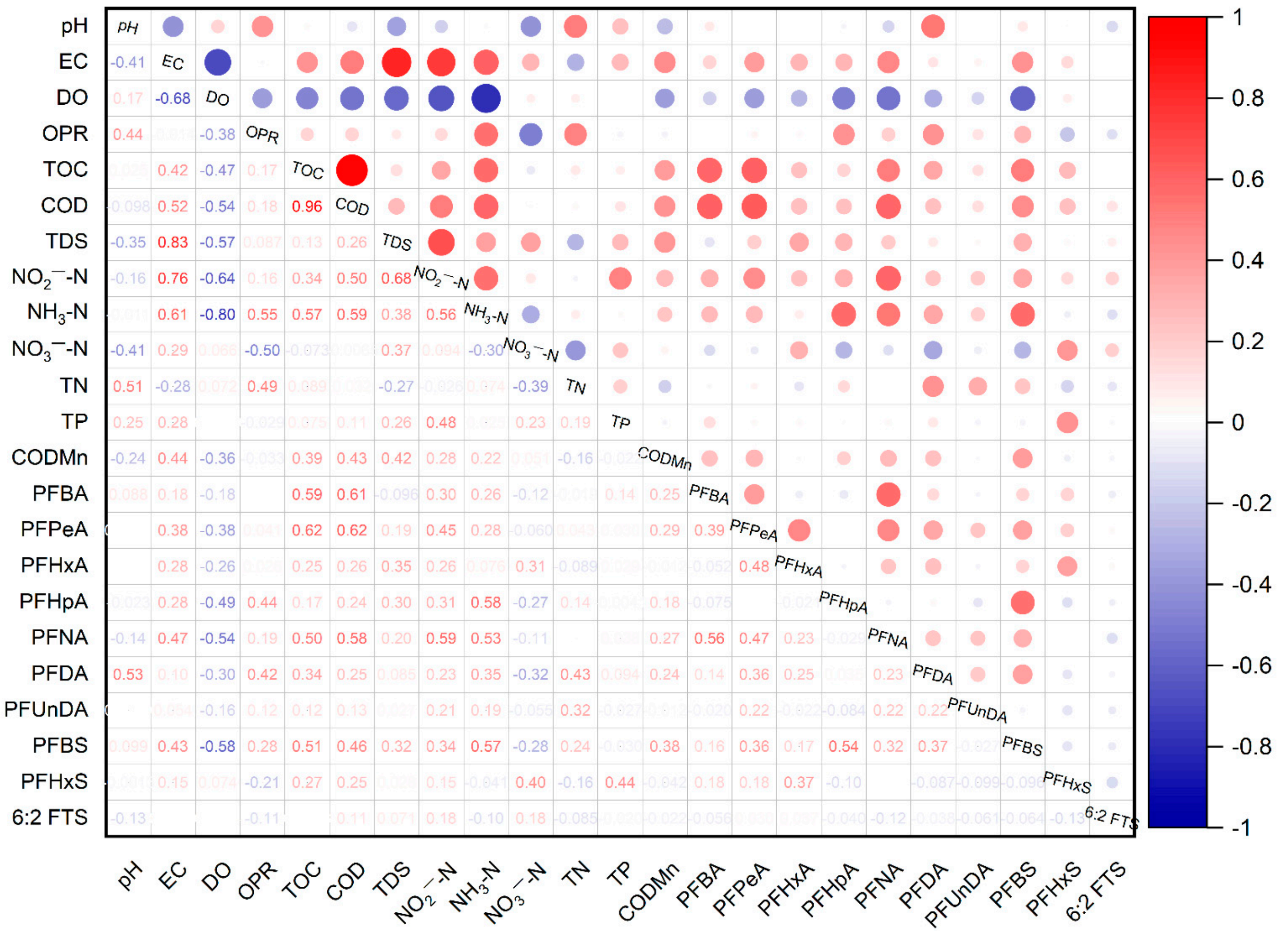

Figure S2 Heat map of pearson correlation between PFAS and water quality parameters in river water

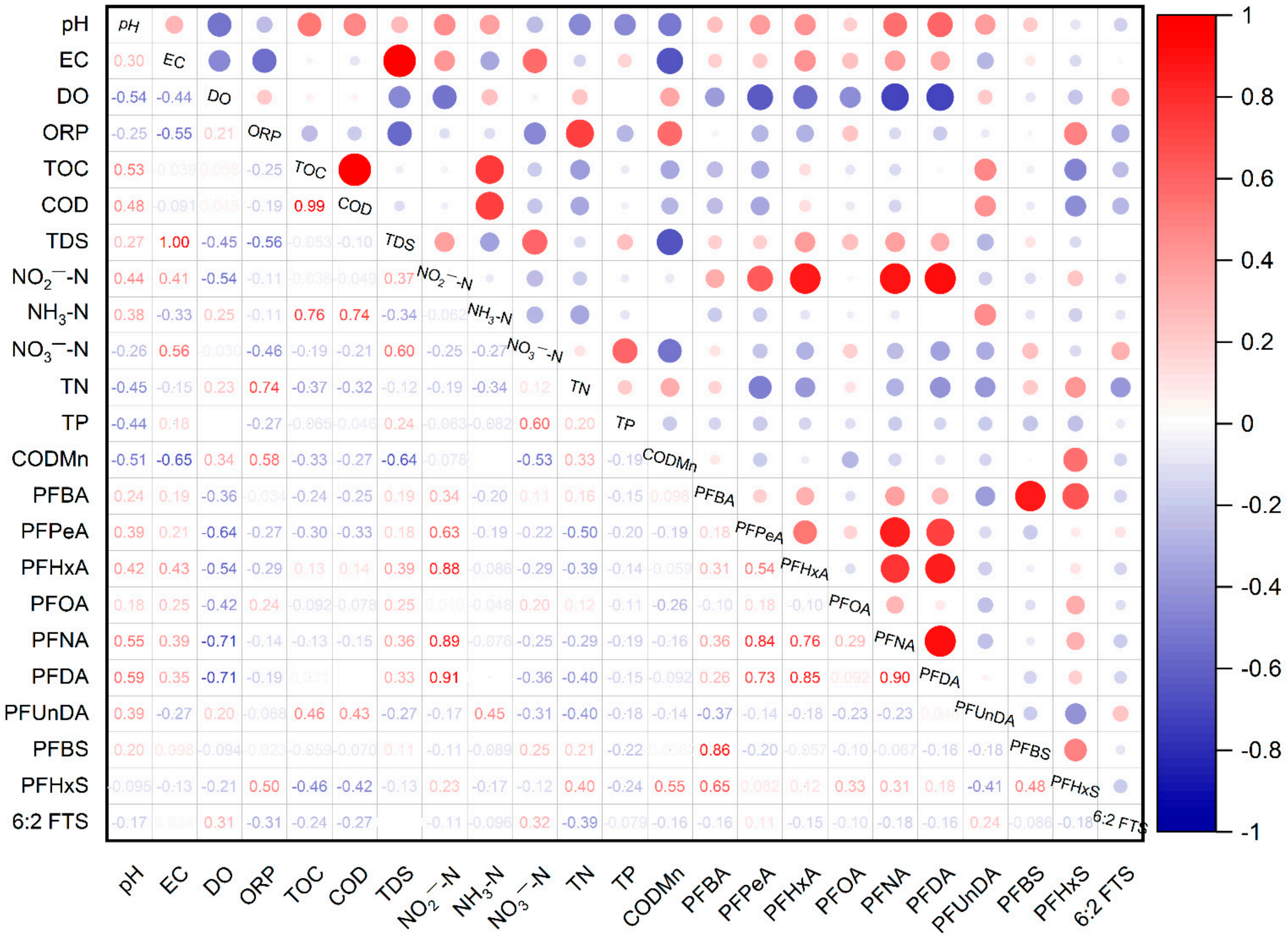

Figure S3 Heat map of Pearson correlation between PFAS and water quality parameters in groundwater

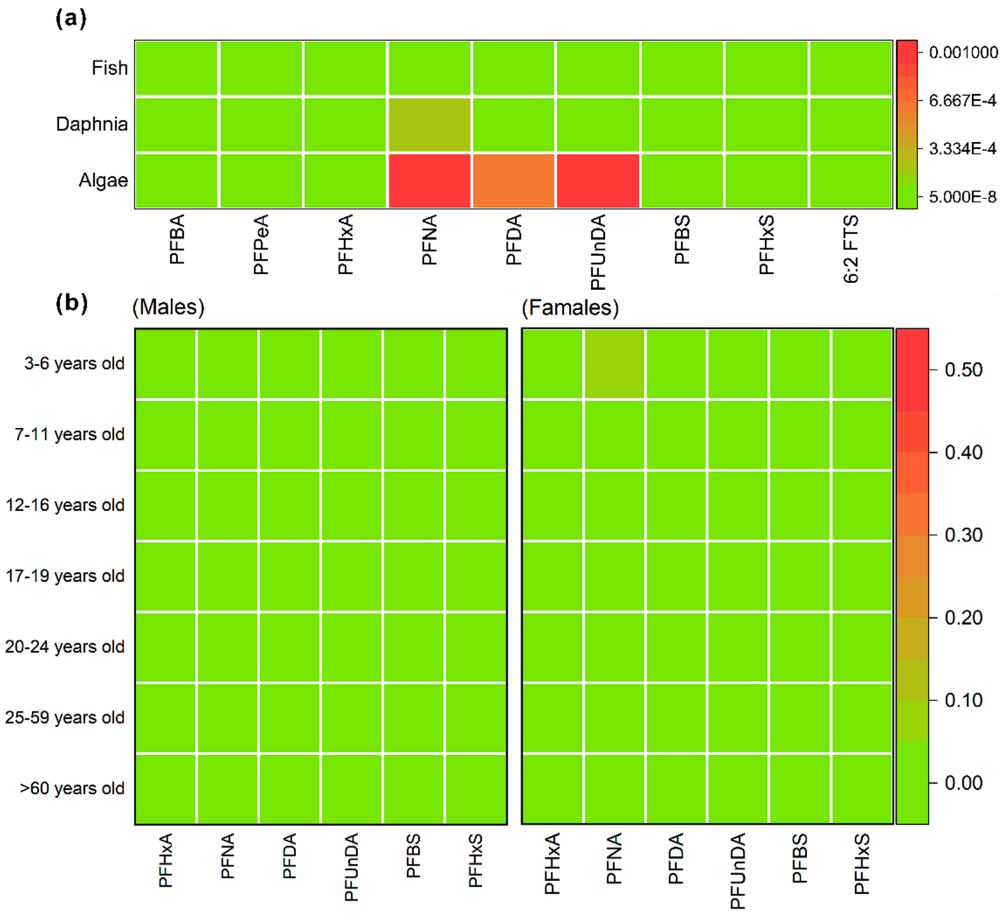

Figure S4 (a) Entropy of risk to algae, daphnia and fish for each PFAS in river water; (b) Health risk of each PFAS in groundwater for different age groups and genders

References

1. Hoke, R.A.; Ferrell, B.D.; Ryan, T.; Sloman, T.L.; Green, J.W.; Nabb, D.L.; Mingoia, R.; Buck, R.C.; Korzeniowski, S.H. Aquatic Hazard, Bioaccumulation and Screening Risk Assessment for 6:2 Fluorotelomer Sulfonate. *Chemosphere* **2015**, *128*, 258–265, doi:10.1016/j.chemosphere.2015.01.033.
